# Co-designing a community pharmacy pharmacogenomics testing service in the UK

**DOI:** 10.1186/s12913-022-07730-y

**Published:** 2022-03-22

**Authors:** Tim Rendell, Julie Barnett, David Wright

**Affiliations:** 1 University of Bath, Claverton Down, Bath, BA2 7AY UK; 2 University of Leicester, University Rd, Leicester, LE1 7RH England

**Keywords:** Barriers, Enablers, Pharmacogenetics, Reflexive thematic analysis

## Abstract

**Introduction:**

Pharmacogenomics (PGx) testing services have been delivered through community pharmacies across the globe, though not yet in the UK. This paper is reporting a focus group study, the first stage of a participatory co-design process to increase the chance of a successful implementation of a PGx service through community pharmacy in the UK.

**Aim:**

To identify the barriers and enablers to implementing a community pharmacy based PGx service in the UK.

**Method:**

Three focus groups were conducted with community pharmacists (*n* = 10), prescribers (*n* = 8) and patients (*n* = 8) in England. The focus groups were recorded, transcribed and thematically analysed using the Braun and Clarke six phase reflexive thematic analysis approach.

**Results:**

The analysis identified five themes about PGx testing in community pharmacies: (1) In- principle receptiveness, (2) Appreciation of the benefits, (3) Lack of implementation resources (4) Ambiguity about implications for implementation and (5) Interprofessional relationship challenges.

**Conclusion:**

The identified enablers for implementation of a PGx service were at a macro health system strategic level; the concerns were more at a granular operational procedural level. Overall receptiveness was noted by all three participant groups, and both prescriber and pharmacist groups appreciated the potential benefits for patients and the healthcare system. Prior to implementation in the UK, there is a need to disambiguate health professional’s concerns of the guidance, resources, and knowledge required to set up and deliver the service and to resolve patient concerns about the nature of genomics.

**Supplementary Information:**

The online version contains supplementary material available at 10.1186/s12913-022-07730-y.

## Background

Pharmacogenomics (PGx) reduces the probability of adverse drug reactions (ADRs) and increases the likelihood of selecting the most appropriate dose. ADRs are reported to account for 6.5% of all admissions to hospital and estimated to cost the UK NHS £466 M annually [1]. As a result, the prevalence of trial and error prescribing should be reduced and patient outcomes enhanced [2]. In line with other diagnostic testing the cost of this new technology is rapidly reducing [3]. To date, PGx testing services in community pharmacy have been implemented and evaluated in the USA [4–9], Canada [10–12] and the Netherlands [13]. Given the precedents set elsewhere and developments in the arena of genomics over the last decade, there is an opportunity to design and implement a PGx testing service for patients in a community pharmacy setting in the UK. Such a service would require dovetailing with the nature of UK health systems and the working practices of healthcare professionals, as well as meeting the expectations of patients as service users.

**Fig. 1:**
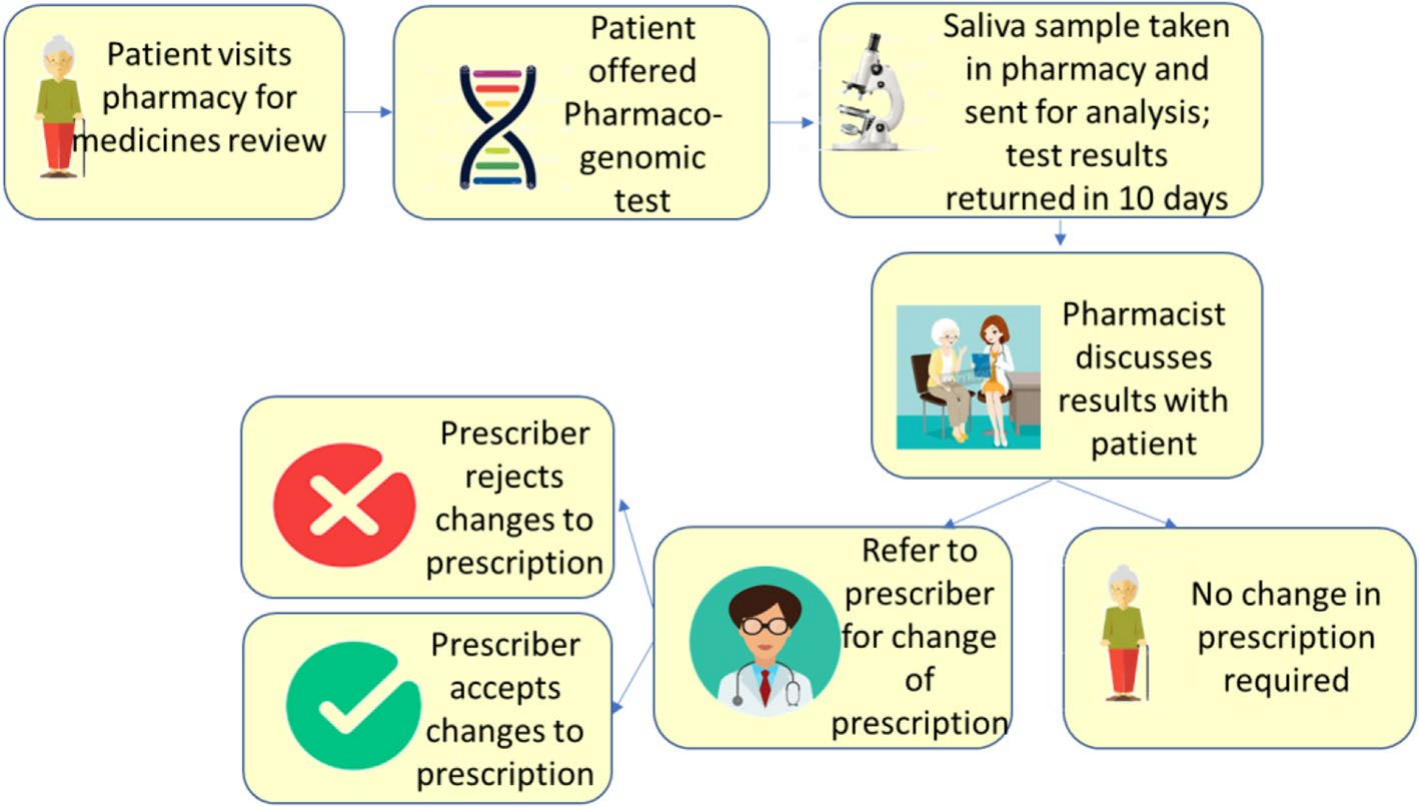
Patient journey for PGx testing service

The PGx testing service in community pharmacy involves the patient taking a DNA cheek swab and sending it to a testing laboratory. Upon receipt of the results, the community pharmacist can review the patient’s medications and make recommendations to their prescriber to amend their prescription, as illustrated in Fig. 1. It is likely that such a service would initially be privately funded and subsequently regionally, then nationally commissioned as an NHS service.

### Community Pharmacy

Over the last 15 years, community pharmacy in the UK has evolved from dispensing of medicines to the introduction of pharmacist led services including the New Medicine Service (NMS), an Influenza Vaccination Service [14] Minor Ailment Services  [15] and Healthy Living Advice through the Healthy Living Pharmacy (HLP) project [16]. There is already a commitment to develop this further with the agreement by NHS England and NHS Improvement (NHSE&I) on a five-year financial settlement for community pharmacy including the introduction of the Community Pharmacist Consultation Service (CPCS), a Hepatitis C testing service, a service for detection of undiagnosed cardiovascular disease and a new service to improve access to palliative care medicines [17].

In terms of the most appropriate setting for pharmacy services to be delivered, Hindi, Schafheutle and Jacobs [18] reported that the access and convenience of community pharmacies was preferable compared with General Practitioner (GP) practices for pharmacy services including “management of minor ailments, medication reviews and routine check-ups for well managed long-term conditions”. Furthermore, community pharmacists are the last healthcare professional to see a patient before they start a new medicine and are therefore ideally located to provide a PGx testing service. By 2025, the new NHS genomics medicine service will be integrated into routine care [19]. Pharmacists could be an integral part of this service, from administering the test, explaining test results to patients, to alerting prescribers to significant gene-drug interactions. However, the exact interface with community pharmacists is so far unclear, but with ease of access and their extended role, community pharmacy has the potential to be the most conveniently located solution for pharmacogenomic testing.

### Scope for a PGx service

A number of small-scale evaluations of community pharmacy PGx services have demonstrated significant opportunities to optimise treatment, afforded by multiple gene PGx testing and at the point of prescription initiation. A recent study reviewing prescription data in the Netherlands estimated that 24% of all newly prescribed medicines for a range of 45 common drugs dispensed in a community pharmacy setting had a potential drug—gene interaction (DGI) [20]. In an evaluation of a PGx service, also in the Netherlands, 17.8% patients were advised to change their drug and 14.0% to amend the dose of their current medication; it was noted that “pre-emptive analysis of genotyped patients showed that the majority (99.2%) had actionable variants” [21]. Similarly, in a recent UK study, it was estimated that between 19.1% and 21.1% of all newly prescribed medicines for 56 drugs involved an actionable DGI, which would have resulted in a recommended change of drug or dose for 8.6%–9.1% patients [22]. Finally, in an implementation study with patient participants (*n* = 100) in two community pharmacies in Toronto, Canada, a descriptive study reported on a range of interventions that required prescriber referral following receipt of a PGx test result. The pharmacist recommended a change of drug for 60% patients (*n* = 41), a dosage change for 13% (*n* = 9), increasing monitoring for 22% (*n* = 15) and stopping the medicine altogether for 4% (*n* = 3).

### Co-design process

This focus group study is part of a qualitative participatory co-design process. Co-design methodology in healthcare research has been found to engage with all the participants, allowing them to “express their creativity and … to articulate the root of the clinical problems” [23]. It can help to identify, “tension between the professional agenda driven primarily by cost-effectiveness and the patient agenda that prioritises the process of care” [24]. It can be helpful in bridging any gaps in the understandings of an issue held by healthcare professionals and patients, supporting shared learning [25]. Participatory co-design has been widely used in healthcare research [26–28], “however, co-design has been infrequently employed in the pharmacy setting, despite the potential convenience” [29].

A recent scoping review reported on learning from experiences of PGx implementation in community pharmacies in other parts of the world, and concluded that”patient interest, pharmacist engagement, training and supporting information for pharmacists and prescriber acceptance of recommendations for any changes to patients’ prescriptions” are all required for successful outcomes [30]. There is evidence about the engagement of each of these three key stakeholder groups with PGx services.

### Patients

Patients as a stakeholder group have also demonstrated an interest in PGx testing. In a study (*n* = 18) in a community pharmacy setting in North Carolina, USA, most were interested in learning more about PGx [4]. In a multiple setting study at five community pharmacies in the same state, 81% (*n* = 56) patients consented to testing with 95% pharmacists (*n* = 53) believing that their patients had understood the subsequent results [5]. Prescribing medicines that work better results in better patient engagement with resultant improved care for the patient as “supporting patients to be actively involved in their own care, treatment and support can improve outcomes and experience for patients, and potentially yield efficiency savings for the system” [31]. PGx facilitates increased engagement moving patients from simply having a list of drugs on a prescription to having a conversation that enables a deeper understanding of which medicines work best for them in relation to their genetic constitution.

### Pharmacists

Overall, whilst there are several practical considerations around implementation, the relevant literature suggests that community pharmacists believe PGx testing to be a part of the evolution of their role. This was noted in a study in Pennsylvania and Ohio, USA where it was reported that “most community pharmacists held positive views about the clinical utility of PGx” [32]. It was also reported that pharmacists were willing to integrate this into their professional practice, having an overall positive attitude to a PGx service [33]. However, the lack of education of pharmacists in PGx has been identified as a barrier to implementation of a testing service in a community pharmacy setting. In a study conducted in Pittsburgh, USA, pharmacists (*n* = 11) “recognised the limitations of their formal education” however “recognized their needs for enriched knowledge and instruction” and noted the required training to be similar to that for the introduction of certified training vaccination programmes [9]. Similarly, in another study in Kentucky, USA, 38% of pharmacist participants (*n* = 101) stated that they had no current knowledge of personalized medicine, however 81% agreed that they would be willing to complete this education programme in the future [34].

### Prescribers

For prescriber stakeholders there were no identified studies reporting interest in a community pharmacy PGx service. However, prescribers were noted to be receptive to a community pharmacy testing service; in a study in a single community pharmacy setting in North Carolina, USA, it was reported that “prescriber acceptance was exceptional for this new service” [4]. Furthermore, in a multiphase study in Ontario, Canada where pharmacists (*n* = 21) delivered the PGx service to 85 patients who had been newly prescribed an antipsychotic or antidepressant medicine, changes to prescription were recommended in 40% cases. Prescriber acceptance of pharmacist recommendations for a change to the prescription was reported to be 68% [12].

### Summary

Whilst multiple gene PGx testing is being developed and delivered through community pharmacies in a number of countries, it is not currently available either privately or through the health system in the UK. There is also an absence of evidence in the literature of a co-design process for implementation of a PGx testing service in the reported studies. The lack of a co-design focus surrounding community pharmacy services, the scope and opportunity for a PGx service, and what has been identified in the literature about the aligned and conflicted perspectives of each of the participant groups suggests that it is important that any service design processes engage with these three key stakeholders at the outset and fully take their views into account.

### Aim of research

To identify the barriers and enablers to implementing a community pharmacy based PGx service in the UK.

## Methods

### Study context and design

The study used a cross sectional design with qualitative methods. Three focus groups were conducted with community pharmacists (*n* = 10), prescribers, (*n* = 8), and patients (*n* = 8). The groups were not mixed to allow participants to comfortably contribute within their own professional and lay groups.

### Study participants, sampling and recruitment

The sampling strategy was to include pharmacist participants who would deliver the PGx testing service (all employees of Day Lewis Plc, a UK based chain comprising 270 pharmacies) and two other key stakeholders in the service, prescribers (GPs in the UK) and patients. Expressions of interest were sought through personal invitations; a purposive sample was used based on an interest in novel healthcare services and the researchers “expert knowledge of the population to select in a non-random manner a sample of elements that represents a cross-section of the population” [35]. As part of the reflexivity of the researcher during the research, to avoid any power conflicts or possible coercion, the pharmacist participants were recruited for the focus group by the Day Lewis Pharmacy Superintendent, by posting an expression of interest on the company’s intranet in addition to a group email to all 332 employed pharmacists. The GPs were recruited by the researcher sending an email to 23 GPs, working in practices co- located to a Day Lewis pharmacy, requesting expression of interest from themselves or a colleague. The patients were recruited by Day Lewis Plc participating pharmacists and Regional Pharmacist Managers who identified patients who had personally participated in a private (not NHS) pharmacy service, for example a Patient Group Direction (PGD).

The three focus groups were noted to have had a good mix of diverse characteristics (Table 1).

**Table 1:**
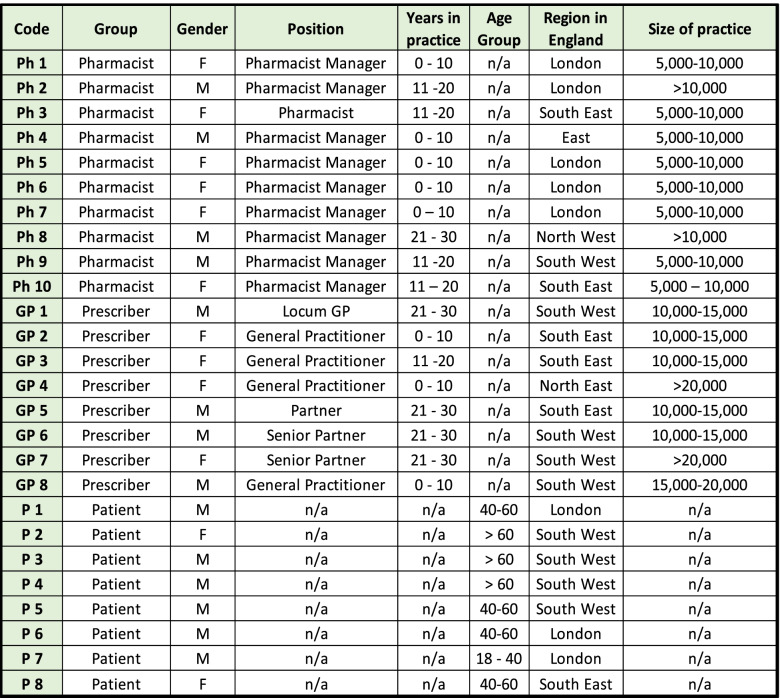
Characteristics of participants in focus groups

*Prescribers = patient list size, Pharmacists = monthly medicines dispensed

### Data collection

Data was collected between December 2020 and March 2021. Online meetings were convened to comply with social distancing requirements during the Covid-19 pandemic [36]. Confirmed details of time, date and an invitation to a Microsoft® Teams meeting was communicated to participants in advance along with consent forms and participant information sheets. Focus group schedules were prepared and used as a guide for questioning the participants.

Prior to the first focus group, a mock session was held to pilot the questions and to ensure the operability and connectivity of the technology; as a result, the questions were modified for clarity and to capture richer qualitative data.

Each focus group was led by a facilitator and an assistant to support timekeeping and recording of data, both having received relevant training. Consistent with the reflexive methodological approach, it was important to the researchers that the design did not stray into a deductive approach, methods used did not force data collection into pre-determined constructs or domains and for the researchers to remain open to the possibilities that an interpretive stance to research demands. In practice this meant using open and semi-structured questions for data collection that were not determined by specific theory or theoretical frameworks, comprising a mixture of semi structured prompting questions and probing questions. Participants were provided with a short briefing statement about personalised medicine and pharmacogenomics, and this was read out and presented at the beginning of the three focus groups to give them an introductory background to the topic. This included the potential patient journey and an example PGx test report. The collected data from the three focus groups was transcribed by an external company, into an orthographic transcript, including anonymisation of all participants.

### Data analysis

This data was then inductively analysed by the researcher using reflexive thematic analysis to identify codes and themes. Specifically, the Braun and Clarke six phase approach [37, 38] was used for the researcher to take a reflexive approach to the data, consistent with the pragmatic and action orientated epistemological approach underpinning this co-design study.

The first phase was familiarisation with the data, with multiple readings of the full transcripts. For the second phase an open and organic coding process was undertaken. The next phase was generating initial themes from the codes, identifying the significance and meaning of the coded data. The fourth phase was to review the themes followed by the fifth phase to define and name the themes. The final phase involved writing up the findings, using relevant literature to add context to the completed thematic analysis. The lead researcher analysed the data using reflexive thematic analysis and the codes and themes were reviewed, discussed and amended over several months with oversight from the co-authors (JB and DW) to discuss, check and reflect on the codes and themes to ensure that the thematic analysis was reflexive.

### Ethics approval

This study protocol gained approval from the NHS Health Research Authority (20/HRA/4147) for the prescriber group. For the pharmacist and patient groups ethical approval was given by The Research Ethics Approval Committee for Health (EP 19/20 069) at the University of Bath.

## Results

The data analysis identified five key themes that pertained to the design and implementation of PGx testing service in community pharmacy, namely:

1. In principle receptiveness
2. Appreciation of the benefits
3. Lack of implementation resources
4. Ambiguity about implications for implementation
5. Interprofessional relationship challenges

These five themes will be presented and discussed with illustrative quotations using the following codes: GP participant = GP, Pharmacist participant = Ph, Patient participant = P and the corresponding number relates to the participants’ unique code in the focus group that they participated in.

### Theme 1: In principle receptiveness

“I think it’s good. I think it’s futuristic. I think it’s innovative. I think it will really help patients” (Ph5).

Although PGx was a new concept to almost all participants, overall, in all three groups, there was a positive receptiveness to the early implementation of a PGx service in a community pharmacy setting.

The receptiveness of community pharmacists was based on their competence as experts in medicines optimisation and a desire to be at the leading edge of implementation of this technological innovation. They recognised the limitations of the traditional “one size fits all “approach to prescribing and instinctively envisaged the potential benefits of personalised medicine in reduced side effects and reduced hospitalisation for their patients.

I think it’s good. I think it’s futuristic. I think it’s innovative. I think it will really help patients, um, not only identify what they should and shouldn’t be taking, but also provide a more individual approach to what they are taking … the first thought I had was, “That is an absolutely fantastic idea”. It’s going to save both the patient and the NHS a lot of – well, a lot of money and also a lot of care. So, yeah, I think it’s great (Ph 5).

GPs also considered accommodating PGx testing as a natural evolution of their role and an additional tool in their armoury in medicines optimisation and management, that resonated with both their professional practise and direct feedback from their patients. They saw it as a potentially supportive tool in their prescribing to address the frustrations of patients who felt that the medicines they had been prescribed were not having the intended effect.

It's something that I've not really heard about before, um, and I think definitely it's really interesting. And I think patients will really enjoy getting their results back. They might feel quite vindicated, I think, in certain cases, to have a look [laughs] (GP 4).

The foundation of patients’ receptiveness was the twin recognition that their current prescriptions are based on a “one size fits all” approach and that PGx would enable their prescription to be tailored to the optimal medication and dose for them as an individual.

It allows medical professionals to uh to-to examine that data and bring that information together that we all sort of desperately need, really (P 5).

Although there was recognition and support for the benefits of PGx, patients also expressed concerns around future imagined PGx related scenarios relating to data security and the identification of future disease states including the potential impact on insurance premiums.

Yeah, just thinking about it, I mean, initially, when you said it, I thought, ‘oh wow’, you know, ‘yeah, that is a great idea’, and then you think about it a bit deeper. And I’m still all for it, cos everybody else brought up the issues that I was thinking, you know, ‘oh, security and to do with insurance’ and things like that. But yeah, I think if I was given the option of it, I would have it done cos I think it’s a great idea (P 8).

### Theme 2: Appreciation of the benefits

“Not only will it help with polypharmacy, it will help with hospitalisation and reducing the cost to the NHS” (Ph4).

Moving on from an in-principle receptiveness, clear claims of the benefits of PGx testing were made by pharmacist and GP participant groups in two areas, for the NHS and for patients.

If a patient needs to take three medicines to control their blood pressure, but it turns out that two of them don’t work for that patient at all because of their genetics, reducing them to one… benefit to the NHS, benefit to the patient (Ph 3).

Both pharmacist and GP groups noted that there would be benefits for the NHS. This would result firstly from patients being more proactively involved in their medication management and optimisation taking part in the discussion that the pharmacist would have with them about their personalised test results. Secondly, there would be cost savings from the avoidance of waste caused by the return, and the subsequent disposal of medicines that have not had the desired therapeutic effect. To guarantee the integrity of the medicines supply chain, any medicine that has left the pharmacy premises may not later be dispensed for other patients as the efficacy, quality and safety cannot be assured, and therefore must be destroyed [39].

We are chucking codeine at a lot of our elderly and perhaps we're just getting a lot of side effects but none of the good effects (GP 3).

Another pharmacist participant also noted the benefit to the NHS of reducing polypharmacy, and the resultant hospitalisation that can result.

The second theme articulated the benefits for patient’s personal healthcare. One pharmacist participant referred to the NHS commissioned New Medicines Service (NMS), where pharmacists offer support and advice to patients taking a new medicine for certain long term conditions [40].

I have a number of patients who I have done NMSs on, one NMS a month for three months, because they’ve been trying out their medication and it didn’t work, it didn’t work, it didn’t work. The idea that they could skip all those intermediate steps and go from one that – basically just go to one that does work. They would be so relieved because people don’t like being messed about. They don’t like trying everything out all the time. If you’ve got an option to jump straight to the right answer, they’re going to be keen on it (Ph 3).

In some cases, it may require amendments to a patient’s prescription before the optimum treatment can be identified; the opportunity for patients to be prescribed the correct medicine first time was clearly welcomed. The benefits of linking the NMS and PGx testing to improve patients’ healthcare were noted and, pharmacists were starting to envisage how the PGx service could be linked to and integrated with existing community pharmacy services.

Our job is to improve patient understanding, um, so we can use that to develop or improve patients’ health literacy. But also, what we can do is maybe integrate this within, uh, within other services in the pharmacy such as NMS. So, while we’re doing the NMS, if a medication’s not suitable, we could say, “OK, because you’ve tried these maybe let’s do this testing to see whether we can shed some more light on what’s more suitable for you” (Ph 4).

### Theme 3: Lack of implementation resources

“So, it can be quite challenging, especially if you’re running a very busy pharmacy” (Ph 2).

Lack of resources to deliver the service was a clear concern identified by pharmacists and to a lesser extent by GPs, as a barrier to implementation. Three specific concerns were noted: the lack of time for healthcare professionals, lack of space and a quality clinical environment in the community pharmacy to undertake the consultation with the patient and a lack of NHS drugs budget should the PGx analysis recommend a more expensive drug.

The concern about limited time to deliver the service was raised by both pharmacist and GP participants noting how the perceived increase in workload would impact their already stretched roles. Such a concern is not unexpected, particularly in a post pandemic environment, however the expression of these concerns was also accompanied by evidence of a willingness to speculate as to what practical arrangements could be put in place to mitigate this.

There’s a lot of challenges within the pharmacy, and just to add another service on top of that is going to really press you in for time, but then again if you can delegate the tasks effectively, then the pharmacy may have a technician, for example, that can take care of the dispensing and the checking (Ph 4).

A couple of GPs were also concerned as to whether patient awareness of PGx and subsequent queries about the appropriateness of prescriptions might add additional time pressures to their workload with delays ensuing in cases where PGx testing was not indicated.

Concerns were expressed by pharmacist participants about the appropriateness of conducting the testing in the consultation room of a community pharmacy.

Some pharmacies, they struggle to carry out flu jabs because of not enough space. There’s hardly enough space to do an MUR (Medicines Use Review). You’re sat not two metres away from somebody discussing their medicines, and they – if you brought out genome testing to them, they’d probably think, “Why on earth have they said that? I’m in this really poxy room with them right now, and how are they going to carry this out?” It doesn’t, it doesn’t seem like it’s fit, it doesn’t seem like it’s a provision that will be appropriate (Ph 5).

The final concern, raised exclusively by GPs, was that pharmacists may recommend a more expensive drug, or one that is not currently within their local NHS formulary.

I suppose one of my concerns would be perhaps that the patient would then get a list of medications that actually were very expensive or that we, we, you know, weren't on our formulary (GP 4).

### Theme 4: Ambiguity about implications for implementation

“Genetics is almost alien to me now, because my genetics training was over 30 years ago” (GP 7).

In addition to the participants in principle receptiveness, appreciation of the benefits and having a lack of resources for implementation, there was a clear theme surrounding the ambiguity encompassing the guidance and knowledge that will be required to design and deliver the service. Despite this uncertainty, all three participant focus groups shared their ideas and suggestions about how the service could be designed and implemented from their viewpoint, drawing on their knowledge of current systems and practices.

Recommendations on how the service should be designed locally within healthcare systems were discussed and GP participants encouraged consultation with Primary Care Networks (PCN) and local GPs champions.

Maybe that might even be a way of generating some funding to try to make more efficient prescribing (GP 1).

Both pharmacist and GP participants argued that the service should be implemented gradually, with a small number of patients and limited conditions, to enable the clinicians to gain confidence in delivering the service and for fear of them becoming overwhelmed. There were also shared concerns that a major launch across multiple settings for all patients and several conditions could be counterproductive initially whilst pharmacists and prescribers familiarised themselves with the testing service.

So, soft launch for everyone to get used to it and, and to make sure that it works and that communication lines are in, and just some gentle suggesting before it becomes a, you know, a big poster in the window saying get your pharmacogenomics done here (GP 7).

Another uncertain element was how results should be stored, both location and format. When asked where the test results should be stored, for example on GP computer system records, in the pharmacy patient medication record (PMR) or by the patient themselves, there was some consensus by patients that the record should be held by their GP, along with all their other health and medical records, and at the same time accessible to the pharmacist. In principle, this is how the NHS summary care record (SCR) works whereby the community pharmacist has full “read” access to all patients’ health records, although currently no “write” access [41].

And as far as I’m concerned, that – you know, the-the doctor should keep hold of that, I don’t – shouldn’t have to carry it around in my breast pocket (P 1).

In addition, there was significant input from all the participants concerning how to design the content, language and format of the results. Both GP and patient participants were concerned about receiving complex information and both requested focussed and concise data.

So, I think that probably if you gave us the information in a relatively simple – either number needed to treat or percentage efficacy – then actually, that probably would, would be all the information we needed, um, as long as it was specific for that condition (GP 1).

There were also some doubt as to when in the patient pathway would be most appropriate to conduct the testing; there was some consensus with both the pharmacist and GP participants linking it to the prescribing of a new medicine, and in particular when the pharmacist conducts the NHS commissioned New Medicines Service [40].

I think that'd be a perfect time to do it, because if you're starting a new medication and you're planning on them reviewing the impact of that in a, in a few weeks' time, having that information at your – at that point - particularly, you know, I can imagine if you are starting an antidepressant drug and the patient's coming back to you and says that hasn't done anything - that would be a great time to know whether that's, uh, a biochemical issue or whether it's a diagnostic issue, or you know, whether you've selected the, the right, uh, treatment modality at all (GP 5).

Perhaps unsurprisingly, in line with current narratives about the pressure on the NHS and its relationship with private providers, all participants were concerned about the cost benefit of the service, with further uncertainty if patients would self-fund and its affordability if it were a private service, and if not commissioned by the NHS, could it lead to further increasing health inequalities?

The cost for me is, is quite important. Who’s going to pay for the test? (Ph 10).

In addition, several concerns were raised by the GP participants regarding doubts about the accuracy of the test, as they were unsure about the sensitivity and specificity.

Um, what are the stats behind it? If it's 100% that, that gene sequence means that that medication won't work and this one will, or is it 50% this one will and the other one won't? (GP 8).

GP participants suggested knowledge they would need and information that they would require to support pharmacists delivering the service, focussing on clarity of the information that the pharmacist would be providing them.

I'd probably want to know for that specific medication, what other medications would you be looking at as alternatives to the ones that that patient can't have. Because, you know, we talk about GPs, we do trial and error medicine, and a lot of the time I'll just start a patient on a medication, they'd feedback it's not working; we'll try another one (GP 8).

Patients also requested some background information knowledge to dispel myths, particularly surrounding the security of their data, and to give them confidence in the service.

I think the first thing in my experience of-of data-related issues is, the first thing people are going to want confidence is their data is protected (P 6).

Furthermore, pharmacists acknowledged the importance of clear and concise information for their patients, considering any health literacy challenges.

I think patients’ understanding of the science is a huge hurdle. Um, you know, enzymes that help you process different medications and how that’s linked to your genes and how that links to what medicines are more suitable, is quite a scientifically complex thing to try and explain to people. Um, and then it’s kind of how the tests would be done with the patients once they’ve kind of crossed that (Ph 9).

Finally, both pharmacist and GP participants raised concerns about whether they had appropriate knowledge for effective clinical decision making, many of whom had not had any training since graduating from University or as part of any post graduate education.

I'm of an era where my, you know, genetics is almost alien to me now, because my genetics training [laughs] was, was, was over 30 years ago when I had any formal genetics training … and I think a lot of us probably don't have huge, huge knowledge base on that, so I think it's probably quite, quite a wide range of training that we'd need to make us feel comfortable in it (GP 7).

### Theme 5: Interprofessional relationship challenges

“GPs love more information. I don’t see any issues with this at all” (Ph 3).

Delivery of a PGx service requires a new or additional pattern of interprofessional interactions between the prescriber and the pharmacist. There were several areas of concern about this. Firstly, there was a concern from pharmacist participants that their suggestion of PGx testing may be considered by patients as undermining their GP. The pharmacist participants were suggesting that the drug or dose prescribed by the GP may need to be adjusted could be experienced as very threatening to long established relationships of trust between patient and doctor.

I’ve had patients walk in and say, “The doctor told me to get this” and you’re looking at them and going, “No, that’s really not the best idea for you,” but the doctor told them and they’re going to stick with what the doctor said. I normally find with my patients that is normally those in the over 70s, over 80s category (P 3).

This potential area of concern was not mentioned by GPs and patients.

Secondly, several challenges to a good working relationship between the pharmacist and prescriber were identified. For the service to be a success for the patient, the relationship between the pharmacist and GP to work in partnership, to collaboratively deliver this service, is essential.

I can see that being a-an issue both ways, one of the GPs feeling the pharmacists are stealing some of their roles; likewise, the, the pharmacist potentially being paid for work that the GPs have already been paid to offer, and the NHS turning round and saying actually, you shouldn't be being paid for this (GP 1).

Broadly the GP participants were supportive of receiving recommendations for a change in their patients’ prescription. However, GP participants drew on other areas of interaction with pharmacists as the context for their concern that recommendations from the pharmacist are made in a constructive manner, that is with a suggestion for a clear alternative rather than coming with a problem, not a proposed solution.

It, it's easy to accept that kind of recommendation as long as it comes in a constructive way. So, we're very used to getting dozens of contacts from pharmacists saying, uh, vitamin x capsule isn't available today, please prescribe alternative but [they] don't tell us what the alternative is. That's a, that's a daily, hourly event, you know. Uh, something's out of stock, please prescribe alternative. What have you got; you know? … And then it's very easy to, to respond, I think (GP 5).

Pharmacist participants were equally concerned about effective communication how best to deliver this. Some were anxious approaching GPs with proposed recommendations of treatment for fear of the response.

And it means that you’re going to have to talk to the prescriber and put a case to them to say, “I’ve done the genomics test on your patient, and you’ve prescribed the wrong thing.” Now, think of the relationship between the patient and the GP. You know, that’s a very fraught area. You can’t ring up a GP and say, “You’ve given effectively a medication to someone that’s causing them side effects or is inappropriate.” That’s a very difficult discussion you’re going to have (Ph 8).

Conversely, other pharmacists were more positive and even saw this as an opportunity to build improved clinical relationships within the primary care team. Pharmacists that already have good collaborative working interactions with their GPs may find this conversation easier than those who have maintained a more hierarchical relationship.

It’s more about a liaison, more information feeding back. GPs love more information. I don’t see any issues with this at all (Ph 3).

## Discussion

A summary of the five themes, classified as enablers for or barriers to implementation is illustrated in Table 2.

**Table 2:** Summary of themes categorised by enablers for and barriers to implementation

Enablers		Barriers		
**In principle receptiveness**	**Appreciation of the benefits**	**Lack of implementation resources**	**Ambiguity about implications for implementation**	**Interprofessional relationship challenges**

### Enablers for implementation

The in-principle receptiveness by all three stakeholder groups serves as an initial encouragement for implementing a PGx testing into UK pharmacy practice and a sound foundation on which the co-design of this healthcare service can be built. This is not a clear green light to go ahead however, the in-principle buy in was at a high level and largely decoupled from considerations of service delivery processes. Furthermore, receptiveness was not unconditional; in particular patients defaulting to data and privacy concerns. Any design of a PGx service needs to robustly address these concerns to remove any barriers to implementation as any issue, however small, could make the difference between a patient agreeing to participate in the service or not. This receptiveness was also reported in previous research [4, 5] with patients showing an interest in the service, high levels of participation and an overall comprehension of their test results. Pharmacist’s willingness and confidence to deliver the service was also reported [33] although interestingly GPs could also see PGx testing as part of their role. This could be a legacy view though as more testing and vaccination services are now undertaken by community pharmacists as part of their extended clinical role.

The healthcare professional groups showed an appreciation of the benefits for the NHS and for patients; a clear enabler for establishing a PGx testing service. There were perceived benefits, mainly from pharmacists to the introduction of a PGx testing service in community pharmacy, ranging from system wide benefits for the NHS to individual patient benefit in terms of improved health literacy, taking responsibility for their own healthcare and improved outcomes. Interestingly, there was a lack of concrete benefits noted by GPs and an absence of benefits noted by patients; this is important to note as part of any service design.

### Barriers to implementation

Understandably, the lack of resources for implementation in a post pandemic world was highlighted and was identified as a barrier to implementation in several areas: time, quality space and budgets. It is important that careful attention is paid to each of these areas and that solutions, whether complete or partial, are co-designed in partnership with those delivering the service. Previous research has indicated that time may not be a significant barrier suggesting that minimal time is required to deliver the service [5] and that it is comparable to a vaccination service which pharmacists regularly deliver as part of their role [11]. Similarly, space requirements for delivering a PGx testing service has been considered as the “least significant barriers” to implementation of a PGx service in a community pharmacy setting [34], although no evidence about the quality of the clinical environment for PGx testing was mentioned. Most community pharmacies in the UK have invested in an appropriate consultation room in recent years that may be suitable for conducting this service [42]. The perceptions of the pharmacist participants differ however, and it will be important to take this into account and understand what their perception of the shortfall in space provision is due to. Finally, limited drug budget resource within the UK health system is a perennial challenge, however this concern may be mitigated if there is an overall cost saving to the NHS, as anticipated. Certainly, all these concerns need to be fully understood and addressed if pharmacists are to offer the service to patients and GPs are to respond to pharmacist’s requests to amend prescriptions.

The theme ambiguity about implications for implementation was also identified as a barrier to implementation. All three participant groups were both uncertain about many aspects whilst helpfully suggesting ideas. They acknowledged a current lack of knowledge, information and guidance that would be required prior to implementation in a UK community pharmacy setting. Despite this ambiguity, ideas were forthcoming from system level, through to pace of implementation, identification of patients, storage and format of test results and the optimum stage in the patient pathway to intervene. Encouragingly, there was some consensus between the three participant groups, despite them all having a different professional or lay perspective on the service design. Pharmacist and GPs self-confessed lack of genomic knowledge was a clear concern. Pharmacists need to have access to PGx knowledge training and gain expertise in interpreting the test results so that they can make appropriate clinical decisions. The literature also reported this as a concern with a recent scoping review reporting that “there is clearly a need to educate the profession and a need to consider if the training is included in the undergraduate programme or whether it is provided when pharmacists commence providing the service” [30]. Consideration will also be required to support GPs with a basic level of PGx training. One particular concern raised by both GP and patient participants was “what else will the testing reveal and what impact will this have on my insurance?”. It needs to be clearly stated that the testing will only report on specific drug-gene interactions. In addition, the UK Government and the Association of British Insurers (ABI) have agreed a voluntary code of practice on the role of genetic testing in insurance [43]. Some of this lack of guidance was also noted in the literature with a paucity of relevant patient information in a user-friendly format reported [4].

The final theme generated from the data was interprofessional relationship challenges, a further barrier to implementation. The literature notes pharmacists reporting high levels of prescriber acceptance of recommendations for an amendment to the prescription [4], the exception being where the prescriber did not have knowledge about PGx testing [10]. With all healthcare roles evolving, and with pharmacists delivering more healthcare services e.g. medicine reviews, hypertension case finding, vaccination services and health checks [44], there was some evidence of potential conflict both in the literature [45] and in the GP and pharmacist participant groups. The advent of pharmacist independent prescribers, with all newly qualified pharmacists gaining this qualification, will also allow pharmacists to make changes to medication in collaboration with GPs, but without having to seek direct approval. There is clearly a potential for Pharmacist and GP interprofessional relationships to be a barrier, and this must be carefully considered as part of the service design.

In summary, the literature reporting on the implementation of PGx testing in other parts of the world resonate with the five themes generated using the reflective thematic analysis of data from the three participant focus groups. This is encouraging and can form the basis of codesigning a PGx service in a community pharmacy setting in the UK.

### Limitations of the study

Although there were a diverse group of participants from across the three key stakeholder groups, the numbers were small and thus the authors offer the findings and conclusions drawn as indicative and open to being supplemented by further research in this area.

It should be noted that none of the participants had any significant knowledge of PGx and none had participated in a testing service, thus their knowledge was limited to their personal experiences in other areas of healthcare and their views may be different if they had experiential practice. Whilst this is not a significant concern, the verbal briefing that was given to participants at the beginning of the focus groups may however have influenced the context for the discussion; indeed, a different perspective may have created a different backdrop to the discussion. Additionally, the study was completed in a small multiple group pharmacy chain. Community pharmacists practising in other settings in the UK may have different experiences and views, and wider research would be required to capture these ahead of developing and implementing a PGx service in those settings.

### Implications for practice

The implications of the co-design and generation of themes from the focus groups mean that there is a real opportunity for the community pharmacy sector to consider implementation of a PGx testing service in a community pharmacy setting in the UK. There is a clear correlation of the outcomes of this study with the literature published outside the UK and therefore the results from this study could be developed to enable implementation of this service in the future. There is now an opportunity to continue utilising the participatory co-design approach in combination with behavioural science to identify behaviour change techniques [46] to operationalise this novel healthcare service, through the development of a service specification for a pharmacist led PGx testing service. This window of opportunity is open and holds promise for the benefit of both patients and the NHS.

## Conclusion

Having completed a co-design using Braun and Clarke’s six phase reflexive thematic analysis, five themes have been generated, the first two as enablers for and the other three as barriers to implementation.

In conclusion, a clear story that has been generated from the data obtained from the three participant groups, categorised as barriers to and enablers for implementation and reinforced by research studies that have been completed outside of the UK. Evidence of a foundational level of receptiveness was noted by patient, pharmacist and prescriber groups, and both professional groups appreciated potential benefits for their patients and the healthcare system. The ambiguity surrounding the service for all three groups needs further clarification at a granular level as part of the service design. Finally building collaborative relationships with prescribers and pharmacists is a challenge that requires careful consideration to ensure the benefits of this novel service to be realised.

## Supplementary Information

**Additional file 1.**

## Data Availability

The datasets generated and/or analysed during the current study are publicly available in the supporting file, in a machine-readable format.

## Abbreviations

ADR: Adverse Drug Reaction
DGI: Drug—Gene Interaction
GP: General Practitioner
MUR: Medicines Use Review
NMS: New Medicines Service
P: Patient
Ph: Pharmacist
PMR: Patient Medication Record
PGx: Pharmacogenomics
SCR: Summary Care Record
