# Supplementary material for: Co-designing a community pharmacy pharmacogenomics testing service in the UK
Source: BMC Health Serv Res. 2022 Mar 22;22:378. doi: 10.1186/s12913-022-07730-y (PMC8939480; doi:10.1186/s12913-022-07730-y)
Supplement: Supplementary file 1 — Additional file 1. [file 12913_2022_7730_MOESM1_ESM.docx]

**GP focus group transcript**

GP-Focus–Group–Novel–Pharmacy–Service–Design 20210113

Facilitator: [00:00:06] I'm just going to do a very brief introduction about what tonight is about, but then the main part is, is listening to your views. So I'm going to share with you some background on an innovative health area of healthcare that we're working on, as starting with a really short briefing on personalised medicine, and then following, the reasons for this focus group which [00:00:35] is about pharmacogenomic testing.

Now, personalised medicine, as you know, is the combined use of genetic, environmental, lifestyle, clinical and other unique patient factors to influence prescribing. And for decades now, patients have been treated with medicines based on our own personal characteristics but assuming that all – they're all average and when we're prescribing, we prescribe personalised for reasons, reasons [00:01:07] such as age or weight, um, other medicines, uh, pregnancy, hepatic and renal disease, etc.

What's different now is the potential to use genomics to improve this prescribing further using standard doses, dosage regimes from formularies, but with the advent of the lower cost of DNA testing it's now going to be possible for more people to have a genomic test and then be able to [00:01:37] target the medicines according to, to the genomics. Now, pharmacogenomics itself is a specialism of personalised medicine, and uses the genomic information to predict an individual's responses to drugs and, as a result, prescribers can then select the best medicine for the patient.

And this could deliver personalised medicine rather than the traditional one-size-fits-all approach. Pharmacogenomics itself is derived from the word [00:02:07] pharmacology, which is the study of drugs, and genomics the study of genes. And as you know, patients metabolise and eliminate drugs at different rates, resulting in some patients needing a higher dose, some patients needing a lower dose, and some patients needing a different medicine altogether.

And this all contributes to the 30% to 50% of patients not taking their medicine, as intended, particularly [00:02:37] for long-term conditions, often resulting in unnecessary hospital admissions. Now, by 2025, the new NHS Genomics Medicine Service, its planned it will be integrated into routine healthcare and pharmacists, and will be an integral part of this service in explaining the test results patients through alerting prescribers on significant gene/drug pair interactions.

Now, to date, pharmacogenomic testing has [00:03:08] been piloted in a range of GP settings, hospitals and community pharmacies in some parts of the world, not yet in the UK. And if I can just show you the sort of patient journey it could look like, or it looks like in some settings in the rest of the world. so, you know, Facilitator 2, can you tell me if you can see the slides, please?

Facilitator 2: Yes, I can see them.

Facilitator: [00:03:35] Right. So effectively, um, the patient comes into the pharmacy for medicines. It may be a new medicine service and then the patient is offered the pharmacogenomic test, which is a cheek swab. The the swab goes off to the lab and then about 10 days later, um, the medicines report is returned to the pharmacist. The pharmacist then discusses the results with the patient, and then there's two options.

Either there's no change in prescription required or they refer it to the prescriber [00:04:05] for a change of prescription, at which point the prescriber either accepts the change or rejects that change. And I thought it'd just be useful just to give you an idea of what a report could look like. So this is for a test patient, so for the five drugs that the patient is on there, you can obviously see there's a significant result of three of the five drugs.

And if then go particularly to the coding at the top there, you can see with that particular gene, the significant result may result in [00:04:35] altering the medication. And therefore, the pharmacist, down at the bottom here, can read the recommendation, so he can see that there's greatly reduced metabolism of the codeine into morphine, so it also says that there's no point giving tramadol or oxycodone, but the gene CYP2D6 is not affected for morphine or buprenorphine, so therefore the pharmacist [00:05:05] could make the recommendation for the prescriber to change from a codeine, um, to one of those two.

So that's – can we just come out of that for a moment? So that's just really a very, very top line of what the service could look like for the patient and then what the outcome. But I guess I'm going to leave this for a moment and really now ask you, having seen this, what are your first thoughts on this new technology, please? Yeah, GP 3, please.

GP 3: [00:05:37] Yeah, no, I, I don't know very much about it, but I had read about it and I think the two drugs that I had sort of read could be useful to use this with was opiates or codeine, morphine, and omeprazole. But I think you mentioned esomeprazole, but I think they're maybe the only two drugs that I've – and that's going to be my question, actually – which common drugs would this, um, really kind of matter in clinical practice? 'Cause it's all well and good with lots of drugs that you may not use very frequently.

Um, I think, you know, we are chucking [00:06:07] codeine at a lot of our elderly and perhaps we're just getting a lot of side effects but none of the good effects. Um, so I think, uh, certainly for – for certain patient categories, and to avoid wasting medication that isn't going to do them any good, it might be really useful.

Facilitator: Yes.

GP 3: [00:06:24] Um, but of course, it – to, to me, it would really depend on which drug will make a difference and, and, and of course the cost of the test as well.

Facilitator: [00:06:33] Yeah, great. Just for information, in, in the US, there's about 100 drugs that are now registered with the FDA, um, with the recommendations on there. Uh, GP 5, please?

GP 5: [00:06:47] I-I was just going to say, um, I do quite a lot of diabetes, uh, work at my practice, and we already take selections of drugs - particularly second, second line - based on, uh, I think it's phenotypes, you know. We know that if people are overweight, for instance, then we're going to, uh, select a drug that helps with weight loss. And this seems like an extension of that that's just another tool to help us make that more tailored decision [00:07:17].

So I think I-I would really welcome this, because I-I see it as a natural progression to the way that we're working anyway. I think, um, the option to, to know about people's tolerance to opiates is really interesting. You know, we could start using that information today, if it was available. But I'm interested to hear what the cost is as well.

Facilitator: [00:07:41] Yeah, great, thank you. So just as very top line, 10 years ago it was £1 million. Uh, by 2015, it was down to about £1,000. Today it's, it's somewhere between about £100 and £200, or for the whole genome, which the NHS - it's about £300. But the cost is dropping all the time, and by 2025, you know, could it be as low as, as, you know, tens of pounds? Uh, GP 7, please?

GP 7: [00:08:07] Yeah, and I think we've probably all got a list of those patients who can't tolerate anything. Um, they've got a long list of anti-hypersensitive to the – doing weird things that aren't, aren't obviously allergic reactions. Um, how useful is it for that? I can see a use for those sort of people who are really struggling to get conditions managed properly, because we're, we're just swapping and changing and everything seems to be a problem.

Facilitator: Yes, yeah. OK, thank you. GP 2?

GP 2: [00:08:16] Um, yeah, I think it looks really interesting. The, um, the thing about the opiates particularly, where it says, uh, tramadol is going to be useless as well but actually these ones would, would work, would be really handy, um, just the more information it can give you, the better.

Facilitator: OK, thank you. GP 6?

GP 6: [00:08:56] Yeah, I'm of the same feeling as GP 7. I think those patients who are struggling with anti-hypersensitive either side-effects or actually managing to get their condition under control, it'd be really useful to know which drugs. We already know that Afro-Caribbeans don’t respond to, but are there other drugs that other phonotypes don’t respond to? It would be useful to know.

Facilitator: Well, thank you. And GP 4?

GP 4: [00:09:20] Um, yeah, I agree. It's something that I've not really heard about before, um, and I think definitely it's really interesting. And I think patients will really enjoy getting their results back. They might feel quite vindicated, I think, in certain cases, to have a look [laughs].

Um, the only thing, my only reservation from just hearing about this is just imagining getting bombarded with lots and lots of these requests to chest… check, uh, change medications, and then having to process those. And I think it would probably involve a discussion with the patient, so I suppose it's just thinking about [00:09:51] how we would manage that in practice.

Facilitator: Yes, thank you. And just to – a number on what you just said, it's 5% of patients, uh, can't break down codeine at all. They just keep taking higher and higher doses. That's interesting. GP 9, please?

GP 9: [00:10:06] Um, I-I think I reflect the same feelings as everyone else, that it sounds like a very useful tool to have, uh, in addition to all the other tools that we do have already. Um, it's, it – I – will it …? Obviously, it's in its infancy in terms of, of rolling it out to, to the general public, but will there be a-a way that we can utilise that in General Practice? So will it becoming directly from the pharmacies to us to say these medications need to be looked at?

Will there [00:10:35] be a kind of a-an interaction so we can feed back for help to get these tests from Primary Care? Um, that's kind of a – it, it works both ways, I guess, um, so it'd be quite useful to know what else this test works for. Um, 'cause I, I've not looked into it that much, so this is quite a new prospect for me.

Um, and it opens up a bit of a can of worms when you start looking at genetic testing, or genome testing, um, looking specifically at a [00:11:05] small list of drug interactions, but where does that open up the, the, the realm for other testings, you know? Well, while you're there, just check this and that, and am I at risk of breast cancer, am I at risk of this? Um, so it does, yeah, it does – it starts to creep onto a boundary, doesn't it, a little bit?

Facilitator: Yeah, absolutely, yeah. Thank you. GP 3, please?

GP 3: [00:11:30] Yeah, that was going to be exactly my, my next question. Is the genome profile, the result that you get, is it purely about the drug? Or would it give you a list of the patient's risk of Alzheimer's and the patient's risk of cancers and their, their likelihood of becoming addicted if they try cocaine? Because I've, I've seen my, my brother-in-law had this test, and it was very interesting. But of course, it raised a lot more questions than, than answers really.

Facilitator: [00:11:58] Yes, yeah. So just, just answering that, and we purposely didn't want to share in advance tonight about pharmacogenomics, 'cause you know, we didn't want you all having to spend hours reading up about it and trying to become sort of experts on it for the call, purposely.

Um, there is one, one test, effectively, um, can give, um, um, lineage; it can also give about, um, um, a diet, about exercise, about drugs, um, predisposition to future disease states. But when you order the test, you can [00:12:28], um, say you've decided you're only wanting to report on the medicines. And what's happening in other parts of the world is they are only reporting on say 100, uh, drug/gene interactions for exactly the reasons that you've said.

But maybe – and this is just me speculating – maybe in sort of 10, 20 years' time, if every child at birth is given the test, then effectively, um, that goes into their records and therefore that, that becomes very, very useful, um, so it comes – that comes with all the [00:12:57] risks that, that you just said GP 9, a-a moment ago.

OK, let's just move onto a bit more specific then, so what do you think your patients would feel? What, what, what – sorry – what do you think would happen for your patients if the community pharmacist did start, uh, doing this testing, so specifically the community pharmacists did the testing now? Yes, GP 5?

GP 5: [00:13:36] I think there's so – I can think of a group of patients who would be clamouring to [laughs], to have this kind of test done. Um, but they, they might not be the people that I was most interested in having the test done, so I think there could be a mismatch, um, between the people accessing it and the people who would benefit the most from it, I think.

Um, but I think the main hurdle to get over would be the [00:14:06] cost, because people are used to a free service, a free NHS, that they have a sense of entitlement to as well. And, uh, getting over the, the barrier of paying for things is difficult. On the other hand, they tend to value things that they pay for a lot more, so –

Facilitator: [00:14:26] Yes, yeah, yeah. And building on your point about which patients, would patients, um, that are prescribed a new medicine be a-a good target population?

GP 5: [00:14:40] I think that'd be a perfect time to do it, because if you're starting a new medication and you're planning on them reviewing the impact of that in a, in a few weeks' time, having that information at your – at that point - particularly, you know, I can imagine if you are starting an antidepressant drug and the patient's coming back to you and says that hasn't done anything - that would be a great time to know whether that's, uh, a biochemical issue or whether it's a diagnostic issue, or [00:15:10] you know, whether you've selected the, the right, uh, treatment modality at all.

So I-I-I'm entrepreneurial, early adapter, early adopter sort of person, so I'm very excited about the whole idea. I-I can think – I'm thinking of lots of ways that I'd embrace that. Um, and I think the pharmacy is a-a, a fair place to have it, but I don't think it'd be long before people were coming and asking me [00:15:40] to arrange that for them.

Facilitator: [00:15:41] Yeah, 'cause the literature talks a lot about trial-and-error prescribing, which sounds, um, uh, doesn't sound that, that scientific. Can you relate to trial-and-error prescribing?

GP 5: [00:15:53] Well, that's how, that's how we work. I work in a very iterative way, and, and that's how we work in General Practice as well. When we haven't got access to a lot of tests, we've got a-a, you know, a differential diagnosis and we test. Either we do a test to confirm it, or we test a therapy to see if it works. So I really recognise that as a description of how I work.

Facilitator: [00:16:20] OK, so overall positive so far, but what are the concerns and challenges that you would have with your community pharmacist delivering a testing service in their community pharmacy, the concerns and challenges? GP 3, please?

GP 3: [00:16:37] Yeah, I think my, my main concern would be the cost versus cost benefit. And because, you know, I don't know who would pay for it, and, and who would select the patient who, who were appropriate for it. I think everybody loves the test, but actually the key would be to use it only for those patients who, who, who – where it would really make a difference, a bit like we shouldn't be ordering a test unless we have a purpose with it.

Um, so that would be my, my biggest concern, because I think it could very quickly turn into a sort of routine tick box [00:17:07] that every would - everybody would get, even if they are quite happy with their medicines and they're working well, just because the pharmacist perhaps would get used to it, would get excited about it.

And of course, if it became an NHS service, which the pharmacy would get paid for, then of course there could be a perverse incentive to test more and more, even if not clinically, um, necessary. So exciting project, but those would be my concerns.

Facilitator: [00:17:32] Yes, thank you, GP 3. GP 4, please, concerns or challenges? GP 4 might be on mute.

GP 4: [00:17:46] Um, sorry, so, um, I suppose one of my concerns would be perhaps that the patient would then get a list of medications that actually were very expensive or that we, we, you know, weren't on our formulary. Um, and then that could cause a little bit of difficulty. I suppose then the point would be is there any point in them being on the other medication if it's not effective anyway. Um, but, um, yeah, uh, that's one of the issues I could foresee happening.

Facilitator: [00:18:12] Thank you. GP 2?

GP 2: [00:18:15] Yeah, sort of along that point, I guess where the results go. Um, do they tell …? Are they stuck between the pharmacist and the GP, um, or do they go to the patient who then comes with a demand of the pharmacist says I've got to have X, Y or Z? Um, and that's one I want. And also, I'm not sure, not sure if it's going to be paid for by the patient or by healthcare. I don't think you actually said that, or not, and therefore there's an inequality if it's patient funded, how many of them can afford it?

Facilitator: [00:18:47] So, so in terms of payments, I'm going to raise that. Of course, in other parts of the world, there's not the wonderful NHS, and it typically is either insurance, uh, paid, or privately funded. And I guess that, um, the, the intention for this would be is that it would probably start as a private service, but, um, hopefully would be seen as cost beneficial to the NHS, and there are, there's some evidence that it is cost beneficial and therefore could be for appropriate patients, um, an NHS [00:19:17] funded service. But that's probably several years down the line.

GP 1: Can I ask something?

Facilitator: Yeah, sorry, yeah?

GP 1: This is GP 1.

Facilitator: Oh, sorry, GP 1, sorry, I can't see you on the screen, sorry.

GP 1: [00:19:28] Oh, I'm down, I'm hiding down the bottom here somewhere, I think.

Facilitator: Sorry.

GP 1: [00:19:31] That's OK. And I, and I was just saying about the important concern for the patient and what you said is that the, that the, the, that the genomics is run and it actually runs the whole patient genomics, but only reports on certain bits of it. Is that …?

Facilitator: [00:19:46] Sorry, I must explain that. So it, it, it has the potential to run all the, the information, but the lab only reports on what it's asked to be reported on.

GP 1: [00:19:58] Right, so there's potentially a lot of information there that potentially might be useful to the patient that may not be being reported on, and I'm just wondering about the sort of the ethics of that and actually saying if there's an identifiable risk, should we – should there be a way of feeding that back to the patient. And I don't know.

Um, having been involved, having had patients who've come in with other, other private services, um, vascular ultrasounds, to look at their cardiac risk, [00:20:27] and spending a lot of time trying to diffuse the pseudo-science behind it, I'm sort of slightly concerned that the – that, that there may be certain elements of the genomics, which is very definite, like the codeine, for example, but there may be other bits that are much softer that actually give people a-a predisposition to benefiting from a medication or not benefiting.

Facilitator: Yes.

GP 1: [00:20:50] And actually, should we be running those on people who, um …? I-I don't know. I don't know the answer. Um, but it – I-I just envisage potentially quite a lot of work coming our way, because actually as the prescriber we take responsibility.

Facilitator: Yes, yeah.

GP 1: [00:21:08] And so we would have to be informed and understand exactly what those tests are, and the, the, the the benefits and the false, false benefits of the advice. Um, and the – coming from a General Practice point of view, actually having that information integrated into your General Practice system, that if you try to prescribe something a little popup comes, another popup comes up and says just, just to warn you, this person might not respond to this treatment, had we thought of this one?

That, that would, I think from a clinical point of view, that would be brilliant. [00:21:38]. And if that information was sitting back in the system somewhere, would there be a way of importing that back into the, the, the clinical system to, to help guide us looking after our patients? Um –

Facilitator: Yes.

GP 1: [00:21:52] And, and talking to, um, other members of Day Lewis about the possible future of pharmacy, particularly integrated pharmacies within surgeries, it actually – the two serv … two services becoming more streamlined, potentially there's an opportunity there of actually the, the pharmacist within this, the, the surgery premises actually being able to deliver some of this support.

And I think, I think that's a very exciting development, although we're sort of blurring the [00:22:22] line between true NHS and, uh - well, maybe GPs are a, uh, uh, slightly unusual part of the NHS, with a-a pharmacist being paid on a fee per, um, for action basis, converse the old General Practice system to provide everything to all your patients is, is – they're two different pay structures.

And I can see that being a-an issue both ways, one of the GPs feeling the pharmacists are stealing some of their roles; likewise, [00:22:52] the, the pharmacist potentially being paid for work that the GPs have already been paid to offer, and the NHS turning round and saying actually, you shouldn't be being paid for this. Um, I-I don't know how this will go.

Facilitator: Yeah, that's very insightful, thank you. GP 5?

GP 5: [00:23:14] Yeah, I-I have the same concerns as GP 1: did. Uh, I think it was, you said, GP 1, about responsibility for, for this information. We're all used to getting incidental findings in CT scans that one hospital clinic have done and then having to, uh, you know, refer to another department. But, but I also had a bit of concern over confidentiality, uh, about the information as well, because – and I-I think [00:23:44] that's just a slightly old-fashioned view about, uh, being the custodian of the patient's record and the information about them.

I've got no objections to people holding all their own records and information, um, but the more places that you have bits of information, more fragmented that can be. And GP 1:, you were saying the same. If it was integrated into the records, that would be fine. If it's [00:24:13], if it's stuff that is known but you don't know it, that could open up all kinds of, uh, medicolegal issues later on.

Facilitator: Yes, yeah.

GP 5: [00:24:25] You've made a choice of prescribing out of ignorance, which somebody else holds information that says that was a, you know, a bad thing to do.

Facilitator: [00:24:34] Yes, thank you very much for that. So as a GP, I guess then, what would you need to enable …? What would you need to enable a pharmacist-led service to actually take place? So additional information would you personally need?

GP 1: [00:24:54] I think that I would want to know the patient had been fully counselled about what what they're being tested for, and the consequences of having those tests. Um, and that, and that's a huge ballpark, 'cause if a whole genome is available, and that's, that's a job I can't do. It's one, I don't have the knowledge, and two, I don't – certainly don't have the time to discuss that with every patient.

Um, and so, there would need to be some very clear way of informing the patient about the implications of those tests. [00:25:23] If they are held on records, um, and they might have, might have identified some risk factors to the patient, how is that going to affect them for insurance and other life, life, um, events? Um, so there's, there's lots and lots of ideas that, that need to be addressed, um, to, to ensure this is done in the patient's best interests, without giving them any detriment.

Facilitator: [00:25:50] And if the report only reports on the medicines that the patient is taking, so in other words, when a test goes off, it reports what, what medicines they're on, would that, would that be a different kettle of fish, if it was narrowed down, from an ethical point of view? Or similar concerns?

GP 1: [00:26:06] I think it depends on what that information is. If someone is a non-response to codeine, then I suppose those people are less likely to become – have an addictive personality. I don't know. Um, but actually, there may be something reported on that has a knock-on effect to that person's risk factors elsewhere and actually by doing that test, the patient needs to know that is – that, that there may be that risk and what that risk – what, what that might do to their, their, their health, health scores, if you [00:26:35] want to call it, insurance scores.

Facilitator: Yes.

GP 1: [00:26:39] But actually, they're, they're, they're prob … If, if - on the NHS screening are very good at looking at benefits, risk benefit analysis, and if someone was going through it and saying we will, we will look at the – and all the, all the hyperten … anti-hypertensive drugs, and say when someone's diagnosed with hypertension, you do that screen and that will guide you on treatment, I think that will be really, really useful.

And you could identify it as being beneficial to the patient. If you sent off a screen and it said, righto, we're going to check that [00:27:09] the top 50 common reactions to drugs – and some of these patients will never need those drugs – then I think that's asking for information that's not actually relevant, and you could argue that's not appropriate. Um, whether it's held somewhere in a database that we could call down at a later date if the patient requires that sort of treatment, that, that, that obviously introduces all sorts of data protection and, and data security issues.

But actually, if I see someone with a new condition [00:27:38] and there's something that helps improve my prescribing, I think all of us as GPs would want to know about that. Um, whether the patients would trust this and agree to it is another question. I'm sure there's a-a subgroup who would jump at it and a subgroup who would, who would run a, run a mile.

Facilitator: [00:27:57] That's great, thank you. GP 3, same question to you, so additional information that you would need?

GP 3: [00:28:03] Yeah, I-I think, I think perhaps I'd like to, to know whether that - you were thinking of doing, um, that test on a patient, because I might have additional information that would make me think this is not a good idea, or this is a great idea. Um, because I think as, as the others have said, you know, that there could be some patients who would jump at the chance, but not necessarily want it for the right reasons.

Um, and what was the other thing that I was thinking about? [00:28:33] Was, um, yes, what sort of capacity would you have to then, um, answer and explain the outcome of those tests later? Because again, a bit like if you do the test, you need to have enough knowledge to explain it, rather than referring back to the GP to speak about all these things. Clearly, if it was a simple thing about actually, they're anti-hypertensive they'd be more suitable, that would be a very direct message.

But again, if those test results got extended [00:29:03] to include, for example, you've got a risk of Alzheimer's, you know, then you are really opening up a-a can of worms with regards to patient anxiety, um, and, and, and I guess that, that is a real kind of … We, we wouldn't want to create more problems, um, for, for no benefit.

Facilitator: Yes, great. Thank you, GP 3. Uh, GP 9?

GP 9: [00:29:22] Um, the – your question was what else, what other information we would require before we carry out the test. I'd probably want to know for that specific medication, what other medications would you be looking at as alternatives to the ones that that patient can't have. Because, you know, we talk about GPs, we do trial and error medicine, and a lot of the time I'll just start a patient on a medication, they'd feedback it's not working; we'll try another one.

They feedback it's not working; we try another one. And we may well have exhausted the medications that [00:29:51] are on that list of, of – that bank of medications that's been tested against. Um, so I guess I'd want to know if we're testing for those medications, which ones are in that bank. Because if we've already tried them, then there's no point in the patient having the genomic test.

Facilitator: [00:30:10] And of course, the other thing is that some patients need a higher dose if they are, um, slow metabolisers of, or a lower dose fast metaboliser, so there's also that dose adjustment as well, which is the, the other, the other key thing. OK, um, what about what, what training would you need to answer pharmacists' questions?

So obviously, the pharmacist would need to do, um, quite a lot of training before they set up this service, both in terms of, um, [00:30:39] how to, um, read the results, how to, uh, use those consultation skills to feedback the results, and then how to have appropriate discussions with the prescriber. Um, what training would GPs need, um, to ensure that they could answer any pharmacist's questions? Yeah, GP 7, please?

GP 7: [00:31:09] Yeah, I mean I think, you know, I'm of an era where my, you know, genetics is almost alien into me now, because my genetics training [laughs] was, was, was over 30 years ago when I had any formal genetics training. Um, and I think there's a big learning curve for the things that others have said. GP 3 was talking about, you know, the Alzheimer's risks, and excluding all that, I think we would just need actually, probably the majority of us, quite basic, uh, going back to basics [00:31:39], looking at genetic risk factors.

And I think a lot of us probably don't have huge, huge knowledge base on that, so I think it's probably quite, quite a wide range of training that we'd need to make us feel comfortable in it, particularly if other things are going to be brought up in that.

Facilitator: Yes, yeah.

GP 7: [00:32:00] Looking at, looking at projecting risks, talking about risks, what they actually mean, I think I would find that, you know, I-I would need more knowledge for that.

Facilitator: [00:32:10] Because both pharmacists and GPs in other parts of the world have felt, um, less than confident and competent, uh, when patients are buying self-testing kits off the internet and then just turning up with the results in their hand. And, um, so, you know, I think that's absolutely right.

GP 7: [00:32:26] Yeah, I think it, it's similar to the people who go and buy, you know, they go and do all this food allergy testing. They come back with a huge list. I mean we – I just cannot – I have no idea what that means and interpreting it would be, uh, you would consider perhaps some of the information might be similar to that - crumbs what do I do with this?

Facilitator: Thank you. Um, GP 6?

GP 6: [00:32:47] Yeah, I, I'm just wondering what, um, consultation have you had with the NHS Genetics Service over this introduction. Because it's a fairly scant service down our way in the Southwest, where everybody [breaking up] [unintelligible 00:33:01] on time. Um, and I just wondered what their, their, uh, take on this is.

Facilitator: [00:33:08] Yeah, good, really good question. I've done some work with the NHS Genomics and effectively, they've been working for the last few years on the 100,000 Genome Project, which David Cameron set up, what, five, seven years ago, and are moving towards the – looking at the million. Um, at the moment it's all about rare diseases and, um, and, and cancers.

It hasn't really moved into the pharmacogenomic space yet. That's all doing whole genome sequencing, um, um, [00:33:38] whereas this is, um, sort of more specific in terms of just the, the pharmacogenomics. But the whole genome sequencing for that does cost an awful lot more.

GP 6: [00:33:46] Yes, because I find it quite difficult to understand the letters that come out from the Genetics Service, and if I had to try and translate this sort of information, it's probably going to be a bit nebulous as well in terms of future use of drugs or potential problems with a drug. I'm not sure I would be particularly well placed to interpret this and explain it to a patient.

Facilitator: [00:34:08] Yeah. Certainly. So the idea is that the pharmacist would make contact with yourself or a recommendation for you to either, um, accept or to, to turn down. But if the patient did come to you asking questions, again what sort of information or training would you need to feel competent and confident with the patient? GP 9, please.

GP 9: [00:34:31] I-I-I think I'd want to know how much, um, it – once the testing is done – how accurate that testing is, um, to that gene sequence, to tell me that that drug would definitely work for me. Um, what are the stats behind it? If it's 100% that, that gene sequence means that that medication won't work and this one will, or is it 50% this one will and the other one won't? What are the, yeah, the stats on that is what I'd want to know.

Facilitator: [00:35:00] Yes, great, thank you. Anything else that you would need to know or need training on yourselves before a service was launched?

GP 1: [00:35:10] I think as GP 9 said, that, um – and if, if we know the, the, the, the relative benefit of those tests, it will help us guide us. And I can foresee actually, that when the results are returned to us with those recommendations, it may not necessarily be change this drug to that drug, it may be change from this family to that family.

And the evidence is they're going to get a 50% more likely to respond or a different family, they're 75% more likely to respond [00:35:41]. I suspect that if the, the evidence is presented in, in a statis … statistical way, which we're all used to looking at, um, we can use that in our – and with the patients. And we're very good at describing risk to patients, um, so I think that probably if you gave us the information in a relatively simple – either number needed to treat or percentage efficacy – then actually, that probably would, would be all the information we needed, um, as long as it was specific for that condition.

And I think [00:36:10] I'd be more interested in a condition-based result rather than a specific drug-based result, because you can often – taking hypertension, we've got so many different therapies within that – and you might be saying, well, this, this family's going to work and that one doesn't. But actually, if you don't mention any of the other families, we don't know whether they're better than …

There may be no genetic link that's known yet, but actually it would be nice to say that, that one, we don't, we don't think there's any need – there's nothing precluding [00:36:39] from trying that family. And so, by doing it in that way, you give us the same therapeutic range that we've got, um, but we, we're moving a lot of the uncertainty about the results per se.

Facilitator: [00:36:51] OK, great. So that, I think that's really nicely moves on to the, to the next question, which is, um, if your community pharmacist made contact with you to amend the prescription following a recommendation based on a pharmacogenomic test, um, would you accept it?

GP 1: [00:37:13] I think it would depend on whether the patient's condition is controlled [laughs]. If it's controlled, there's no point in changing [laughs]. But yes is the answer.

If, if you, if you're struggling to control a condition, I think it'd be useful and, on the same stage, actually if we knew those testings were available, particularly if they're at no cost or very low cost to patients, um, then actually it might be that we'd actually recommend patients to get the test, because we're struggling, and, and use it's a way of helping us out of a-a-a-a [00:37:43] a challenging clinical situation, like we did advise patients to go and buy their own blood pressure machines when people got white oak … white coat hypertension.

We're doing at already. It's just a slight extension of that sort of procedure, I guess.

Facilitator: Thank you, GP 1. How difficult will it be to accept or to turn down that recommendation from the pharmacist? GP 5?

GP 5: [00:38:12] It, it's easy to accept that kind of recommendation as long as it comes in a constructive way. So we're very used to getting dozens of contacts from pharmacists saying, uh, vitamin D capsule isn't available today, please prescribe alternative but don't tell us what the alternative is. That's a, that's a daily, hourly event, you know. Uh, something's out of stock, please prescribe alternative. [00:38:42] What have you got, you know? So as long as information comes with a constructive, uh, suggestion, that's fine.

But yeah, don't say to me this patient, uh, won't respond to ACE inhibitors, I need the whole picture, I need to know if they'd respond to a calcium channel blocker; I need both sides of it. And then it's very easy to, to respond, I think. Half, half the time, it's just as easy as sending the patient a text to say I'm changing [00:39:11] your pill.

Facilitator: Yeah, thank you.

GP 5: [00:39:16] It's the way we are working now.

Facilitator: Great. GP 4, please.

GP 4: [00:39:22] Yes, I think that it, it would be easy as long as, again, you know, they didn't get an alternative that was say an amber drug in our formulary. So let's say they recommended to then start mirabegron instead of whatever they've been on previously and then we have to get permission from the hospital or – and, and that turns into a long discussion. And, you know, it might be giving the patient hope that there might be a medication that they can take, but then it takes a bit longer to get that sorted out for them [00:39:51].

Facilitator: Thank you. GP 6?

GP 6: [00:39:54] Yeah, I was going to say, um, we've just incorporated two pharmacists into our, uh, practice and way of working and dealing with patients now for a couple of years. One or two of them, as you know, used to work with you guys. And, um, so we're quite used to [unintelligible – breaking up 00:40:10] provided they are constructive recommendations that are a useful way of interacting sort of thing. We've found that very beneficial over the years, definitely.

Facilitator: [00:40:23] OK, great. Any other comments on sort of the pharmacist's recommendation and, um, your thoughts on how they can best do that? GP 2, please.

GP 2: [00:40:38] Yeah, I think the, um, the – having a whole list of, of everything that's a possibility in all the minor interactions probably wouldn't be as useful, but if you've got sort of red [00:40:46] interaction on that diagram you showed at the start, then those would be the most recent ones where there's benefit or potential for harm, or sort of ineffectiveness. Um, if the patient's just not going to get any benefit but they're going to potentially have the, the side effects as a result, those ones would be the most useful, I think.

Facilitator: OK, thank you. OK, let's move onto the, uh, the next question, which is if we were going to set this, uh, service up in a Day Lewis pharmacy, what advice would you give us?

GP 1: [00:41:25] I would suggest you put, make a presentation to the local GPs first, before, before you start telling the patients [laughs].

Facilitator: [00:41:35] That was my point as well, yeah. GP 7?

GP 7: [00:41:38] I would also do a very soft launch, because I think, you know, particularly we know our patients and we know that there are that cohort who will be queuing to get it done. And so, soft launch for everyone to get used to it and, and to make sure that it works and that communication lines are in, and just some gentle suggesting before it becomes a, you know, a big poster in the window saying get your pharmacogenomics done here.

Facilitator: Thank you. Uh, GP 9?

GP 9: [00:42:14] Um, that, that kind of reflects what I want to say about, um, kind of don't – possibly a pilot targeting specific conditions and, and medications, so we can pick out the patients we want to and run searches ourselves, and just get a feel of as to how it works, how it's fed back, uh, iron out the kinks and, and then, and then start, um, kind of widescale. Because, uh, if you announce it to our population down here, um, our phones will be buzzing nonstop [00:42:44] with I want this genetic test that's happening. Queues will be out the door, um, as they tend to do. So I-I think doing it as a trial would be a good start.

Facilitator: OK, thank you.

GP 1: [00:42:57] And would it worth discussing it with the local CCG prescribing advisor and seeing whether they may want to come onboard to try to identify patients on polypharmacy for certain conditions, to see whether there may be a way of targeting medicines more effectively at those? Maybe that might even be a way of generating some funding to try to make more efficient prescribing.

Facilitator: Yeah, fantastic, yeah. Thank you, GP 1. Now GP 3?

GP 3: [00:43:21] Um, yes, I think, I think, you know, finding a small target or a few different target groups would be really helpful, um, to, you know - perhaps it will be the ones on polypharmacy and the ones on antihypertensives with still poorly controlled blood pressure or something, to really look at small samples to start with, rather than, you know, Mrs Bloggs who's on two drugs and actually is quite happy with it. Um, how, how, um …? I-I don't know how, how real this is [00:43:52], whether this is something Day Lewis has actually, you know, set up to run from next month, or whether this is Utopia. Um, how …?

So you, you mentioned this is going to be a private service for the, for the time being. Would you be looking at, uh …? I mean what, what, exactly what costs would it be to the patient and how would you, how would you sell it to the patient if they have to pay for it themselves? Because I think, you know, I-I-I totally agree with, with some self-funding.

I think that often encourages people [00:44:20] and it will, you know, perhaps choose people who, who are particularly interested, um, in some cases. So how, how do you sell it to patients if it is not a free service, and, and what was my other question? Uh, no, I can't remember what my other question was, it will come to me.

Facilitator: [00:44:37] OK. Let me allay a few of your fears, or one of your fears there. So, so, so many times in my sort of 40-year career, I've seen new NHS services been implemented and then six months later, they are reviewed and evaluated. With this, part of my research project is to design, co-design a new service specification, co-designing with pharmacists, co-designing with, uh, GPs, co-designing with patients, and then once you've got that specification, then [00:45:07] consider implementation.

Um, so this is, this is certainly, um, not next month, I promise you, um, and, and it's about co-designing a service by listening to all the stakeholders first. So I hope that helps. Um –

GP 1: [00:45:23] And in a, in a Utopia, I think it's a nice way to launch it both for GPs' peace of mind, but also target [00:45:31]. We're very aware of patients demanding something, but actually the people demanding may not be the people who actually need it.

Um, and actually, if, if, if you came to us with the service and we could then refer patients to you, so it's not actually directly accessible to the general population, and so we, so we can use it in a targeted fashion for the challenging patients, well, [00:45:52] then that would give us the chance to learn how to use the service.

Um, I accept it's not going to necessarily give you the same financial rewards, because there, there wouldn't be the opening up to everybody, but actually, I think it will be a – to me, it would be a less challenging option. If I had a patient I couldn't control their blood pressure, then I could recommend this test and from that I could then help control it.

Then I think that would be a-a, an ideal system which would fit perfectly within the ethos of the NHS, of giving patients [00:46:23] the care they need rather than the care they want.

Facilitator: [00:46:26] Yeah, absolutely, and thank you, GP 1. Um, GP 7, please.

GP 7: [00:46:29] No, my point was the same as GP 1's, actually. It was targeting and actually thinking about referring into it as a suggestion, so similar, similar point.

Facilitator: [00:46:40] Great, thank you. So anyone got any final thoughts they'd like to, to share, um, over any of the questions that I've covered tonight, so really capture, um, all of your views in terms of designing of this service? GP 7, yeah?

GP 7: [00:46:54] Yeah, just wonder how, how useful it would be in terms of de-prescribing for the - I don't know – perhaps our nursing home patients now who, who were always … You know, I think we, we're not as proactive as we feel, even though I think we're all keen to, to, to doing that. I mean it is actually how useful it would be to help us as a tool in, in removing drugs from people's lists.

Facilitator: [00:47:23] Thank you very much, thank you. GP 5?

GP 5: [00:47:26] I-I was just thinking if you were piloting, uh, such a scheme in an area, it would be good to engage with a-a-an interested clinical, uh, GP, you know, GP lead or some kind of enthusiast or champion. Because rather than launching a service and then expecting 80 GPs suddenly have the time to embrace it and, and engage and educate themselves, you – it – these things usually work better if you have [00:47:57] two or three people that you're going to, uh, test it on and they then become the, uh, the mechanism for spreading it out to their colleagues.

I mean I can imagine – I work in a big surgery and I can imagine, uh, in, in my surgery there would be some people who would always say, oh, I don't know anything about that, you need to ask so-and-so, you know. And you'd naturally have people who were more expert in it [00:48:26] than others. But that's the way things often, uh, organically grow, so that – I think that's usually a good model to roll things out.

Facilitator: [00:48:37] Fantastic, thank you, GP 5. GP 9?

GP 9: [00:48:39] Uh, I'd probably also try and incorporate … I-I agree with, um, picking out those, uh, those single GPs and, and have them be the advert for you. But, um, also the, the pharmacists, so we've, we've got a few, a couple of pharmacists in the pharmacy tech in the practice as well and having them onboard as part of like a little team within the practice.

Um, because in the end, all those changes will probably start to filter through them and then to us at some stage, is how I would think of it [00:49:09] would work in our practice. Um, so having them onboard will help, because all the GPs listen to our pharmacists, like they know everything. Um, so actually, that would be a good, good way to start it, I think.

Facilitator: [00:49:22] Thank you, GP 9. Are they …? Can I ask you, uh, GP 9, are they CCG pharmacists or federation pharmacists, or, or just within the practice?

GP 9: [00:49:33] They're within our practice. We have one PCN pharmacist that does stuff from afar, um, kind of called a PCN. And we employ a pharmacist in our practice fulltime, and we've just employed a pharmacy tech and another one starting soon. But we, we've got 17,000 patients so we kind of need that extra hand.

Facilitator: [00:49:57] Fantastic. Any final comments?

GP 1: [00:49:59] Could I just, just reiterate that, um, if the system does come onboard, that actually the providers of the clinical systems are also in the loop. So any information that's provided, actually automatically gets integrated into prescribing advice, because I think that otherwise it's going to get lost somewhere in the background, and this should be done.

And without knowing exactly where to look for it, you're not going – you may end up prescribing things that patients don't respond to. So they – and suddenly you see – and EMIS has got very good clinical feedback, it's actually having a-an alert flashing up [00:50:30] saying unlikely to respond to. I think it would be really useful.

Facilitator: [00:50:35] Ideally a summary care record as well?

GP 1: [00:50:39] I-I-I suspect it probably ought to be, yes. And it, and that, that's – I hadn't thought of it that far, but – and I-I envisage eventually the clinical systems will all evolve into one, and, and so we'll have everybody's – we'll have access. The patient's record will be one record and different clinicians might have different – access to different levels of that. But actually, that, that prescribing information will be most useful across all prescribers, um, so –

Facilitator: And [overtalking] work, GP 1?

GP 1: I'm retiring next year [laughs] – this year now, actually.

Facilitator: [00:51:18] [Overtalking]. Facilitator 2 and Facilitator 3, is there anything that I've missed this evening?

Facilitator 3: [00:51:28] You were – there was a-a question around, um, medicine optimisation, but actually I think the responses to the last question about utility, um, and things you could do, the opportunities around this, covered that. So, so I feel like quite comfortable with that.

Male Voice: Yeah, fantastic.

Facilitator: [00:51:47] OK, well thank you all so much for joining. Um, my PA will ensure that all of your invoices get paid tomorrow, hopefully, or processed tomorrow. So, um, um, thank you for sending those through as well, but, um, most importantly, thank you so much for giving up an evening in such a busy time at the moment.

And your insight and information has been so invaluable, um, and, um, some has tied up with what's happening around the rest of the world, but you've actually brought, you know, a very unique, uh, UK perspective to this [00:52:17] and I think that's so, so important too. So, uh, thank you very much and one day when the service does launch, wherever it may launch, um, you, you are part of that, and one day soon it may even be a nationally commissioned service, who knows?

GP 3: [00:52:32] Can we get our genomes tested then?

Facilitator: Yeah, fantastic. Thank you so much, everyone. Have a lovely evening.

**Patient focus group transcript**

Patient-Focus–Group–Novel–Pharmacy–Service–Design -20210302

Facilitator: [00:00:05] I’m going to start with a really easy question, just so that we can all sort of get into the flow. The first question is, what are your thoughts about your regular pharmacy? So who’d like to go first? What are your thoughts about your regular pharmacies? OK, Patient 2, let’s start with Patient 2, please.

Patient 2: [00:00:23] We think our – well, I certainly think our pharmacy is just brilliant. We live in a village and we have a little pharmacy literally at the end of our road um with a wonderful pharmacist who’s very good um at advising. And yes, he’s full-time and the opening hours are the usual opening long hours.

Facilitator: [00:00:45] That’s great, thank you Patient 2. Just bear with me two seconds. Hello, Patient 5.

Patient 5: [00:00:57] Hello. Sorry I’m late.

Facilitator: [00:00:58] A big welcome to you. We’ve just started, but can I just ask if you’re – if um you’re happy for us to be recording tonight and have your consent?

Patient 5: [00:01:08] Yeah, that’s fine.

Facilitator: [00:01:09] That’s fantastic. I just went through a few ground rules, but I’m sure you can imagine what – what they are in terms of not talking over each other and such like.

Patient 5: [00:01:18] Yeah.

Facilitator: [00:01:19] Fantastic. And I just asked, what – what are your thoughts of your regular pharmacy. So Patient 2 gave a positive view. Um I’m very happy to have very mixed views, so they don’t all have to be positive, so would – would anyone else like to say what’s their view? OK, Patient 8, please.

Patient 8: [00:01:35] OK. Yeah, again, mine is um – it’s positive. Um it’s that they’re in a-a very small little parade of shops just down the road from us and they’re always very helpful. Um you know, very happy to recommend things. And they deliver local medicines and I have my local f-flu jab there – joo-flu jab there, so um yeah, I think they do a good job.

Facilitator: [00:02:01] Yeah, fantastic. Thank you. To – um Patient 7, did I see your hand was up?

Patient 7: [00:02:05] Yeah. Yeah, um my thought would be positive as well because my pharmacist is really local to me. Also, many of the pharmacists – the gentleman himself – is really friendly because sometimes, like in [unintelligible 00:02:19], I can’t make it, you know, I’m tired and I get their driver to drop it off to me and stuff like that. So it’s a very odd occasion, like, you know, I’ll get him to-to deliver to me, but most likely, like I’d rather to go them because they’re very friendly staff.

Facilitator: [00:02:34] OK, thank you Patient 7. Patient 6, I think I saw your hand up as well?

Patient 6: [00:02:36] Yeah. I mean, I-I think it’s led by the personality you – you know, the pharmacist you have in the shop, and my pharmacist is absolutely superb. I mean he’s – he’s on the ball, he’s very reassuring, he gives consistently good advice – I would say better advice than my own GP actually. Um and I think that – and this is just a – I’m sure we’ll get onto this – I think that it’s just a shame that we don’t follow more of the European model when it comes to pharmacists. That-that’s my view. Um yeah.

Facilitator: [00:03:09] OK, great. Thank you. Anyone else want to – uh yeah, Patient 5, please.

Patient 5: [00:03:14] We’ve-we’ve got a really wonderful pharmacist attached to my surgery, um and that makes a massive difference for us actually, that our – the-the lead pharmacist there, [name] is – she’s on the ball, she knows what she’s doing. She’s built good relationships with us in the surgery, um she sees patients – UTIs – all of our – sort of anything that can be done under a PGD, she’ll see them and sort that out for us. She’s flexible. For example, today she didn’t have any docinab [00:03:48] in, so she organised for it to be brought over for a patient from another surgery in time for us to give the injection that day, by being that flexible.

Facilitator: [00:04:00] OK, thank you. So I think we’ve cert- you know, I think we’ve got some pharmacy likers here tonight, which is always good to – always good to hear. So – so we’ve got a very diverse uh-uh group on here tonight, so right across the country and-and lots-lots of different diversity, which is think is really-really great. So – and I’m hoping for some-some mixed views as well. Now, what I’m going do is, I’m going just-just share with you what I want to talk to you about tonight for about three or four minutes. [00:04:30] But the rest of the time is going be listening to you. So if you think I’m talking at the moment, it’s just really just to introduce a new topic – and I’m going take it fairly slowly, if that’s alright.

I’m going share with you some background details on a new area of healthcare, and it’s called ‘pharmacogenomics’. Now, don’t worry too much about what it’s called, but it’s called ‘pharmacogenomics’. Now, this uses genomic information [00:05:00] from your DNA and looks for small variations within specific genes. So that’s the scientific bit. So what it can do, it can predict your response to drugs, and as a result, can provide the prescriber additional information about selecting the best drug and the best dose for you, as a patient.

And this [00:05:30] moves closer to personalised medicine whereby rather than traditional one-size-fits-all where prescribing decisions are based on what works for an average patient. Because we know that all of us on the call -we breakdown and eliminate drugs at different rates, where some of us will need a higher dose, some of us will need a lower dose and some of us will need a different [00:06:00] medicine altogether. So this is fairly ground-breaking medicine as a result of the new technology of DNA testing.

Now, in summary, we know that some medicines don’t work because your body is not designed to deal with them in the way that an average patient would do. So for some of us, the liver will clear it away quickly and for others of us, [00:06:30] the liver won’t clear it away at all, meaning you’re more likely to have side effects. And now there’s a test which allows us to look at some of your genes and decide in advance which drug is best for you as an individual, or which dose is best for you as an individual.

Now, your local community pharmacist could, in the very near future, offer this service to patients. And [00:07:00] what I’d like to ask you tonight is, what are your views of the idea of pharmacists offering this when normally you would start a new medicine. So I’m just going show you one slide tonight – and it’s only going be one slide I promise I’ll show you – of what this could look like in your community pharmacy. So hopefully you can see a picture on the screen now. So can you see that, Facilitator 2?

Facilitator 2: [00:07:32] Yes, I can, Facilitator.

Facilitator: [00:07:33] Yeah, OK. So what would happen is, if you were prescribed a new medicine from your GP, the pharmacist would offer you a pharmacogenomics test. What that means is that you would have a cotton bud and you’d do a cheek swab. That would go into a test tube in a little bag and be sent off to a laboratory. And then ten days’ later, the test result would then come back to the [00:08:03] pharmacist. The pharmacist would then discuss the result with you and there’s a couple of options.

Either no change in the prescription would be required, or there may be a change in prescription, which may be a change in medicine or a change in dose. The pharmacist would then discuss that with your GP and either the GP would either say, “Yes please, I recommend that change,” or, “No thank you, [00:08:33] keep to the original medicine that was prescribed.” Let me just stop sharing, if I can, for the moment. Have I stopped sharing, Facilitator 2?

Facilitator 2: [00:08:43] Yes.

Facilitator: [00:08:44] Yeah? Great. So I would really appreciate, what are your first thoughts on what I’ve said? OK, let’s start with Patient 7, please. You’re on mute, Patient 7.

Patient 7: [00:09:02] Can you hear me now?

Facilitator: [00:09:03]We can, yeah. Thank you.

Patient 7: [00:09:05] So um I believe it’s a very, very positive step we are taking because it’s not just dur-during the Covid, but in general, getting in touch with your GP and changing medicine or repeating stuff, it takes uh such a long time. So if-if you had the option to go to – go to your local pharmacist, because a pharmacist, um you know, that knows your uh previous records, realise, you know, um whether you need it at that given time. So I believe in terms of like the time continue – uh the time uh steps, it’s a very, very positive steps.

Facilitator: [00:09:41] OK, thank you, Patient 7. Um Patient 1, did I see your hand up? Again, you’re-you’re on mute, Patient 1. Sorry.

Patient 1: [00:09:52] Oh yes. Can you hear me now?

Facilitator: [00:09:54] We can, thank you.

Patient 1: [00:09:55] Um, I mean, in some ways, it sounds like – sounds-sounds really positive. My concern is security, I guess, security of information. Um whether it’s just – is it just checking a gene for a specific thing or is it more looking at your genome generally? Uh, you know, there may be knock-on thing where kind of this may suggest that ‘oh, they may – may be more susceptible to a cancer in the long [00:10:25] run’ or, you know, quite often one gene will play off against another. Um what happens to that information? Would it go to – would insurance companies be able to get hold of it? Um, you know, cos that may have implications, either for your insurance or jobs or whatever. Um so security around the information is a concern for me.

Facilitator: [00:10:48] Yeah, great. Thank you. I’ll just answer a couple of these as we go through. So it’s only testing um a few genes that are relative to the medicines that you’d be taking. Um and it tests for about 50 to 100 medicines, so that’s-that’s the-the first one, yeah, but real-real valid concerns, thank you.

Patient 1: [00:11:07] Uh-huh.

Facilitator: [00:11:08] Yes, Patient 3 now, please.

Patient 3: [00:11:12] Um yes, fascinating. Rather – rather like science fiction stuff, this is, isn’t it? Um my first uh instinctive reaction would be training um of the pharmacists and also, you know, once-once – again, part of the reason for this focus group is because um you’re actually looking also at the uh the actual boundaries between the GP and the pharmacists. Um now to-to actually look at the DNA profiles uh means [unintelligible 00:11:43] and it’s a problem of, would the GPs be actually happy that the pharmacists themselves um would be um – would be trained perhaps over and above what they can offer. Um, I don’t know, but some training, I think, is-is a maj-major issue here. I can see there’s [00:12:12] some benefits of this, um but certainly training and that those boundaries between pharmacist and-and the GP um would have to be really laid down clearly.

Facilitator: [00:12:24] Yeah, I think you raised some really important points there. This is the third focus group. We’ve actually had eight pharmacists in December and eight GPs in January asking very, very similar questions. So I think that the points you make are-are very well made. Can I go to Patient 6 now, please.

Patient 6: [00:12:42] Yeah. As some- as somebody in artificial intelligence, um actually it’s quite interesting. Patient 1 brings up the first question that was – that’s raised with every oil company and every-every merchant bank I speak to – security. And um the answer, generally, comes back is, we’ve all got our bank accounts in the cloud already, so really there’s not a lot to worry about. Security and SSL protocols are there. In terms of rollout, I mean I-I think it’s marvellous. This-this is next- this is – this is something that um that basically is [00:13:12] – I-I think it’s actually a weak link in the system right now, having a third party um analy- third-party analytics of a situation.

And in fact, so I understand, the Israeli system has already got this embedded and that’s how they’ve managed to roll out the COVID vaccine so effectively and so quickly. And actually have those-those custom- those-those patient profiles available for both the pharmacist and the – and the doctors. So that’s why they’ve bay- basically – they’re so far [00:13:42] ahead of everybody else in the world, you know, in-in real terms. So for me, it’s a natural progression of our health service. Really is.

Facilitator: [00:13:50] Yeah, great. So as-as you – as you said, it is in other parts of the world, it’s big in Holland and certainly in parts of America. Um the-the question- the concerns that Patient 1 raised in – about the security and insurance, um very much are being-being looked at. Um but as you know, just because something’s in another part of the world, you have to make sure that, you know, it’s right for the

Patient 6: [00:14:13] – Yeah, sure.

Facilitator: [00:14:13] – the British medicine service and that’s, again, why – things we’re looking at tonight. Yeah, thank you.

Patient 6: [00:14:17] Absolutely.

Facilitator: [00:14:18] Can I go to Patient 8 next, please?

Patient 8: [00:14:21] Um yeah, hi. Yeah, just thinking about it, I mean, initially, when you said it, I thought, ‘oh wow’, you know, ‘yeah, that is a great idea’, and then you think about it a bit deeper. And I’m still all for it, cos everybody else brought up the issues that I was thinking, you know, ‘oh, security and to do with insurance’ and things like that. But yeah, I think if I was given the option of it, I would have it done cos I think it’s a great idea.

Facilitator: [00:14:47] OK, thank you Patient 8. Patient 3, please.

Patient 3: [00:14:51] Yeah, one-one of the-the things that is int- interesting is that both Patient 2 and I have actually lived um within-within Europe for about um 20 years, both in um (Country) and in (Country). And of course, one of the things there that is-is awfully evident is the-the pharmacist is far-far-far more powerful, shall we say, than the actual doctors. And one of the reasons is, is because, of course, doctors, in the main, are all private. [00:15:21] So-so you – so here in England uh we have a sort of slightly different attitude and-and so therefore the NHS um – bless them – and the – and-and these various primary care-care trusts – or whatever they’re actually called now – um they-they have a completely different outlook. And-and hence my concern about-about the bound-boundaries. But the pharmacists out in Europe – far-far stronger, far more – you know, everyone goes to a pharmacist [00:15:51], frankly, because it is – it is actually too expensive to go to a doctor every-every time –

Patient 2: [00:15:56] – Much quicker. –

Patient 3: [00:15:57] – and-and is quicker.

Facilitator: [00:15:58] Great. Thank you. OK, so Patient 5, did you have your hand up? Sorry, yeah. Thank you. You might be on mute, Patient 5?

Patient 5: [00:16:07] I knew I’d do that. Um questions that I was thinking. In-in terms of would it be centrally-funded or would it be something that the patient took on as an additional service. Um in terms of it increasing workload as well, you know, I-I’ve prescribed some amlodipine for some blood pressure and then coming back to review it ten days later, [00:16:37] it’s already looking at, you know – if that swab is being sent off, would that information be gathered for all of the available medicines that we know about the genomics of and that information passed back into primary care? Or would it be – a test for we’re now prescribing for diabetes, we’re now prescribing for blood pressure, we’re prescribing them individually. [00:17:07] Now, are we getting to the point where we’d have to involve them each time?

Facilitator: [00:17:14] Yeah. So the early trials or tests, we’re just doing one – testing one gene for one medicine, but more recently, it’s called apanel where it’s actually testing for a range of genes – not all of them, but a range of them. So that you only have one test in your lifetime so that any new medicines that you have later on in life, you can still go back to that test. And I think you raise an important question about cost, because ten years ago, [00:17:44] tests cost £1 million. Five years ago, it was £1,000 and now it’s down to the hundreds. So by 2025, when this could be integrated in the NHS, it could be down to pounds or tens of pounds, and that’s why this is becoming available fairly quickly because the cost of the technology has massively reduced.

So it’ll probably be a private service initially – [00:18:14] similar to flu. So you remember that flu in community pharmacy was private in 2010, but now it’s an NHS service. So it probably, as it proved the value, it will probably be NHS in the future, would be my prediction, I suppose. If that helps, Patient 5.

Patient 5: [00:18:33] Yeah, it just raises questions to me about two-tier systems and being given information about some patients and the fact that generally the patients that would benefit from this with the – I presume this would be around things like blood pressure medications, diabetic medications. They’d be the-the medicines that you’d be feeding back to us on. These patients generally are patients from lower [00:19:03] socioeconomic backgrounds, so it – this – a few hundred pounds is quite a lot of money to somebody in those situations. As opposed to diseases of the wealthy are less common in that sort of thing.

Facilitator: [00:19:21] Yeah, very well – a point very well made, Patient 5. Absolutely. So let’s now go on to the benefits. So what do you see the benefits to you, as a patient, if you had the test? Yeah, Patient 6, please. You might be on mute.

Patient 5: [00:19:48] Now-now I’ve switched off my camera. There we go. Yeah, they’ve gotta make this easier with voice, haven’t they? Make it automatic. I-I think it’s more-more targeted treatment plans, um getting-getting to the-the hub of the issues much, much quicker. I mean, you know, typically, with me, I – you know, I’ve got asthma, I’ve got – uh I think uh I’ve got hiatus hernia, I’ve got a couple of things going on. And being able to perhaps even, you know, link those-those – you know, those-those – you know, later on when you [00:20:18] can actually bring-bring medicines together, perhaps, for two or three different um underlining [sic] conditions at the same time.

I think if we’ve got that data and we’re churning it and we start to – we’ll find – we’ll find more links um between-between treatments as well. Um so-so I think – I think all-all round, it, you know, it allows – it allows medical professionals to uh to-to examine that data and bring that information together that we wall sort of desperately need, really.

Facilitator: [00:20:46] OK, great. Thank you. Patient 1, please.

Patient 1: [00:20:52] Oh, I needed to unmute myself. Um with this, are you talking about just identifying um the efficacy of drugs or are you talking about identifying potential ummmm ailments? You know, the-the-the propensity to have high blood pressure in later life or any of those things? I mean, I-I’m not sure which you’re talking about – just whether the blood pressure part- the [00:21:22] drug would be effective or whether you’re identifying future possible propensities to an ailment.

Facilitator: [00:21:30] Yes, a really good question. This-this test is just about the medicine that you’re prescribed – is it the right medicine for you? It’s not about future disease states, it’s not about ancestry, it’s not about diet or exercise. It-it’s completely about the medicine that you’re prescribed.

Patient 1: [00:21:50] OK. I mean, it sounds like a really positive thing if they can – if they can make it that the-the medication I’m taking will be geared around the-the best possible medication for me. I can’t think of a downside.

Facilitator: [00:22:05] OK, thank you, Patient 1. Uh Patient 8, did I see your hand up a minute ago?

Patient 8: [00:22:09] Uh yeah. I agree with-with um Patient 1 actually. If um if it is just literally for that one thing, I can’t see a problem with it and I think it is a fantastic idea. Um and I can also see benefits in other ways, if it should, you know, sort of go a little bit more in-depth, you know, even to do with um – I don’t know, like finding-finding donors for things. As long as you give your consent, you know, that people can look through it, then [00:22:39] um it would be great. But yeah, so the actual – what – the specific question that you’re asking, I think it sounds like a very good idea and I would be happy to do that before I took any medication.

Facilitator: [00:22:51] OK, thank you, Patient 8. So shall we – let’s move on to concerns, cos I mean you raised some concerns earlier and I was going pick up now Patient 3’s earlier concern. Because one of the concerns I think you raised, Patient 3, was about, if the pharmacist makes a recommendation to the GP, how might the GP feel. But that-that’s me putting words into your mouth. Do you want to try and just express again what you think – the concern that you had earlier?

Patient 3: [00:23:17] Yeah, I think one of – one of the areas – um and here we talk about bound-boundaries – is [sighs] if you added-add in the-the current pan-pandemic, I’m abs-absolutely convinced that, because we have spent so long now having this sort of socially-distanced G-GP, and the fact that we are – that GPs will-will-will now see that um it is um it is possible to have Zoom, Microsoft Team-type um consultations –

Patient 2: [00:23:56] – And telephone.

Patient 3: [00:23:57] – telephone – yeah. We actually get an awful lot of telephone conversations now, but I think that’ll-that’ll happen more and more. And therefore, the-the pharmacist will be come our point of – point of contact and I think that is – that is the concern. Because uh will-will the GPs want to rel-release their-their power, but-but are they actually – are they being actually forced to do it? Because uh it is now [00:24:27] cheaper to do a telephone conversation um and/or a Zoom – a Zoom consultation rather than having a face-to-face and all the um – you know, having-having – booking your appointments and going on there and waiting in a waiting room, etc, etc. –

Patient 2: [00:24:41] – And it’s the time involved, isn’t it?

Patient 3: [00:24:42] And it’s the time. Uh whereas it is much easier to go into a – in-into a pharmacist, but that then swings the authority for-for um sort of medicine – OK, fine to be begin with, it’s a small number [00:24:58], according to the DNA, but that will increase. Once – if, in fact, this comes in, there will be an increase in the sort of things uh so pharm-pharmacists um will be able – able to do. And will – [00:25:12] and-and will to want to be, because they’re sort of um – they have a sort of union, I suppose, you know. They will – they will want to see, a) more-more-more money being given to the pharmacists, and perhaps, therefore, less to the GPs, because they’re doing less, maybe? I don’t know. Uh but that’s the sort of area.

Facilitator: [00:25:35] OK, thank you, Patient 3. Now Patient 6, please – and you might be on mute, Patient 6.

Patient 6: [00:25:47] Here we go again. I-I totally agree with what Patient 3 said there um 100%, but it’s already happening. I mean, I think there was some – a headline in-in one of the East London papers that one of the – one of the trusts plans to close 30% of GP surgeries and go virtual. So doctors are – and also, this week – this week – this week in the US – and we always follow the US – CVS, Walmart, in-in their pharmacies, they’re all going virtual, OK? So, you know, I think [00:26:17] 80% of the drug dispendents – dispensed in the state of California during the COVID-19 was basically completely virtual. There was no interaction at all.

So I-I-I think that in-in terms of the question, I-I can’t see any negatives because that’s all good for the planet and that’s all good for the ecological – you know, the-the travelling to and from, the eco argument. Um in terms of negative- negatives, I-I-I think, look, it-it’s just something that we’re-we’re on the wave now – the data [00:26:47] wave, the information age – and I don’t think it’s anything – I don’t think it’s something you can stop, quite frankly. Uh I think we’re already there. Um so negatives-wise, um I think – I think – I think that um, you know, it’s-it’s hard to find a negative, in my – in my view.

Facilitator: [00:27:03] OK, thank you. Um sorry, Patient 4, did I see you ask a – any concerns?

Patient 4: [00:27:11] I don’t – um no, I mean, I agree with uh most of what’s been said. Um the doctors – and we seem to be bypassing doctors in a way here. Um it just seems that they’re gonna have so much more information. And so it’s not just about the pharmacists, it’s – you know, the pharmacists – I accept at the moment, and the pharmacists know more about the drugs than the doctors do, quite-quite clearly. Um but the more information the doctors can receive um it’s gotta be a good thing. Um very, very positive.

Facilitator: [00:27:40] OK, thank you, Patient 4. Uh Patient 1?

Patient 1: [00:27:47] Sorry, I just wanna chip in again. Something I can see as an issue? I don’t know. Um potential problem, is, educating GPs. Um while I’m sure a lot of GPs are very up to speed on the stuff you’re talking about, you know, over the years, I have met some terrible GPs that stop learning the day they left uh um – what do you call it? Doctor school [00:28:17]. Um and it – I think they need to be – some GPs maybe need to be educated about the possible advantages um and what-what you’re aiming to do. Um –

Facilitator: [00:28:33] – I mean, I think that’s a fair comment. I think – I think all of us, as healthcare professionals, that medicine is moving every single year, every single month and we all just keep trying to learn and keep up.

Patient 1: [00:28:44] And there’s always gonna be the kind of ‘oh, stuff and nonsense. I’ve been prescribing such and such for 20 years now, you know, I’m not gonna change’. You know, I’ve met some doctors that have given me bad decisions based on either ignorance or very outdated um uh knowledge.

Facilitator: [00:29:04] OK. Thank you very much. That – let’s just imagine now that the pharmacist did offer the service to you in your community pharmacy. What information would you like as a patient um to give you confidence in what was being offered? So what sort of information? Imagine that the – that either they-they gave you some information verbally or gave you information in a leaflet, what information would you like in that leaflet or to be given verbally [00:29:34]? Um and it’s alright to repeat some of the things that you’ve said already. So imagine that we’re now developing a leaflet or – and explaining it to a patient, what should go in there to give you the confidence? OK, Patient 6, please. Sorry, you might be on mute again, Patient 6.

Patient 6: [00:29:55] Uh I think the first thing in my experience of-of data-related issues is, the first thing people are gonna want confidence is their data is protected. They – you know, you’re not gonna get away from that, um and I think it – in a leaflet, you-you really just want to sort of, in layman terms, lay out, you know, w-what this will do for you, moving forward. What are the benefits, how are we gonna target things, how we can – how we can get a – you know, a better result for you without overselling it. But I think that the data is gonna be the first thing on everyone’s lips.

Facilitator: [00:30:28] The data security, yeah.

Patient 6: [00:30:30] Yeah. Yeah. The use of the data and the reuse of the data – they will want to –

Facilitator: [00:30:34] – Yeah, thank you. Patient 2, please.

Patient 2: [00:30:38] Uh I think um that to – because it can be very personal, because it’s um done with gene testing, I think um if that was put across in the publicity, that al-although the-that this is all to do with IT, the – what it’s capable of doing, um people can often think of IT as-as just being the computer and that’s it, and it’s very impersonal. And I think if it was put across as being even more personal, because of – it is so individual because of the facility that you’re offering.

Facilitator: [00:31:11] Thank you, Patient 2. Patient 5, please.

Patient 5: [00:31:20] I think I-I, from a professional point of view, want to include the complexity argument that although, yes, your genetic testing might suggest that you’re gonna respond better to a certain type of drug, I think it would be really important to make clear that the-there’s more to my prescribing decision than just necessarily what drug is gonna work best for you. Um and I think [00:31:50] some – that’s an argument we quite often have now, so I think that that would be something that would really need to be made clear in it. That although this – it might come back and suggest that you’re going to respond to one diuretic better than other, for example, the fact that we’ve chosen a specific diuretic sometimes is in a – in a wider context than just what’s going to be the most effective medication.

Facilitator: [00:32:16] So it’s not just a report coming back, saying, ‘computer says x’ or ‘computer says y’, it’s part of a decision-making with other

Patient 5: [00:32:24] – Yeah.

Facilitator: [00:32:24] – other considerations about the patient.

Patient 5: [00:32:28] Yeah, the-the-the – the complexity of prescribing needs to be present in that information leaflet, that it really isn’t as simple as, I-I sit down and I type something into the computer and pick a drug from a list.

Facilitator: [00:32:44] Yes, yeah.

Patient 5: [00:32:45] But there’s-there’s – and I – I think conveying that would be quite difficult to do because sometimes people underestimate even their own complexities.

Facilitator: [00:32:58] Yeah, thank you, Patient 5. Patient 3, please.

Patient 3: [00:33:03] Um I think the other thing that would be necessary um is um both age and special needs. Um now I’m in fact not too sure what age you can actually do DNA testing-testing from – could it be birth? I-I don’t know, but is it, in fact –

Patient 2: [00:33:22] – It is. –

Patient 3: [00:33:22] – OK. Um but it, in fact, should be actually put – put across that you are able to actually have-have this from whichever age. And-and there is no um sort of final-final age when-when you can’t have sort of DNA taken. And also, what happens with um – with um sort of special needs. Would-would there be – would they have to have special – uh for instance, um D-DBS clearance for so pharmacists to actually sort of handle um vulnerable-vulnerable people and to actually take the tests to then prescribe. Um there is an element there which sort of sends –

Patient 2: – [Unintelligible 00:34:06]. –

Patient 3: – [takes care of them? 00:34:07] as well. I don’t know. Something like that might actually come up.

Facilitator: [00:34:12] Right, thank you. When I first mentioned the word ‘pharmacogenomics’, how did you feel? Patient 2? Sorry, Patient 8, sorry. Patient 8.

Patient 8: [00:34:30] Uh that’s OK. No actually, just-just-just harping back to the last bit, um I-I like that everyone said about making a leaflet or whatever um uh for the layperson and to try and allay fears and tell you what it’s all about and keep it personal. Um but with the – you know, I-I would be worried about the um security side of it as well. And thinking in that way maybe instead of – I don’t know [00:35:00] how you would do it, but instead of like a central database where it all goes to, maybe like your local doctor would hold any information. So that when the – if a computer gets hacked – I don’t know about computers, but um could it not then be – uh it would be harder to hack if they had to go into lots of different doctors surgeries. And then it may be more personal, also, to you and your doctor.

Facilitator: [00:35:28] Yeah, thank you. Thank you, Patient 8.

Patient 8: [00:35:30] But that word you came up with initially, I thought, ‘oh my goodness, what’s that?’

Facilitator: [00:35:34] Yes. Uh Patient 6?

Patient 6: [00:35:39] Yeah, I-I-I think it – I think it needs to be abbreviated and productised a little there. Um I think if you – you know, it will – it will just blow eight-80% of the population’s brains with just the word, to be honest with you. And I think – I think it’s actually quite frightening to-to the – the lay – layperson – uh not the layperson, but the average person. Um what was I gonna say? –

Facilitator: [00:36:01] – So the word is the mixture of ‘pharmacology’ – the study of drugs, and ‘genomics’ – the study of genes. But it’s a horrible big [00:36:08] word.

Patient 6: [00:36:08] Yeah, but maybe it’s uh phigi or phogo – phigi is good, that’ll be attractive, you know. I don’t know. But, you know, it’s like – it’s like everything in technology, you’ve gotta have an al- you know, you’ve gotta – you’ve gotta break it down a little bit and stretch it and make it more attractive. Um where – so that’s actually a really, really question I think Patient 8 just came up with. Where are they looking to store all this data? Is it – is it gonna be U- on UK servers? Are we – do we know all of that yet? Cos that-that obviously plays a really big story in all of this.

Facilitator: [00:36:39] Yeah. So the data is analysed at the lab and then the security of the data around the lab, and there are about um 15 genomic labs around England at the moment. –

Patient 6: [00:36:49] – OK, OK.

Facilitator: [00:36:51] But round the count- around the world, some people have a personal paper genomics passport, if you like. Uh other countries, it’s held on the GPs records, the GP medical uh database. Um all of these things, and that’s, you know, part of the reason for asking, is just to-to get-get your views. But what’s most important is that, wherever it is, it must be completely secure.

Patient 6: [00:37:17] Yeah, no, absolutely. The only – the only thing I would say is, obviously, that you’re-you’re – at the end of the day, the patient will – like it – like with all GDPR laws and everything else, the patient will ultimately um be allowed whether you store – you know, you could consent to store the data or not afterwards anyway. So uh –

Facilitator: [00:37:34] – Yes.

Patient 6: [00:37:36] – I can’t – I can’t see an issue – um I can’t see an issue with that. So yeah, great. Just-just wanted that clarified. But as I say, just-just from my point of view, I think it needs a brief analogy, I think it needs a – it needs to be brought down so um you can just literally turn round and say, ‘we’ve got this new service – phigi – or whatever, and off you go’.

Facilitator: [00:37:55] Thank you. Well Patient 1, I think, so – so I [00:37:57] saw your hand up?

Patient 1: [00:38:01] I-I’m uh the same, you know, a big, long word, and I thought ‘oh my God’, and then I heard the word ‘genome’ in it and thought ‘ah, OK, it’s that’. But li-like most scientific words, they’re always a bit um unapproachable. You know, personalised medicine makes a-a lot more sense to me um rather than ‘pharmagenomics’ [sic] or whatever it was.

Facilitator: [00:38:26] But building on that question, what does – what-what – when I say ‘DNA testing’, what comes to mind?

Patient 1: [00:38:35] Sinister stuff.

Facilitator: [00:38:36] Mmm.

Patient 1: [00:38:38] You know? Um because it’s – DNA testing, to me, is about having my whole genome worked out, which, as I said earlier, was all that stuff about one’s predisposition to cancer, to um all that stuff, which is not what you’re talking about. You know, you’re talking about very specific um things and so I think so the word [00:39:08] ‘genome’ and so on is – for me, misleading, cos it takes me off in that kind of um sinister – somebody’s gonna know all about me.

Facilitator: [00:39:18] – Patient 3? Patient 3, please.

Patient 3: [00:39:23] Uh yes, my automatic reaction is um as – is, in fact, based on, I think, an awful lot of the sort of um science fiction and films which you-you see these days. But um usually um so young-youngsters and obviously people like myself hear the-the word ‘DNA’, you associate it with alteration, you know, people altering the DNA. Like look at what the reaction of the anti-vax people here um who have actually said [00:39:53] that, you know, that you are going to change the DNA by having a COVID jab. Now, that’s been – that’s-that’s been actually sort of talk-talked about, you know. Abs-absolute cods-codswallop, but it shows the path that some people’s minds are actually taking.

And especially young-youngsters who are – who are sort of mixing what they see on social media and on films uh with this idea that, you know, perhaps they are going to be cloned [00:40:23]. Perhaps this is going to in-in-inject a sort of microchip inside them and their DNA is going to be totally altered. You’ve gotta sort get-get-get rid of that idea.

Patient 2: [00:40:35] Yeah, I would say – I initially thought of Dolly the sheep, we’re all gonna be cloned all over the place [laughs].

Facilitator: [00:40:44] Thanks, Patient 2. Patient 6, please.

Patient 6: [00:40:47] Yeah, I-I think that’s why it’s absolutely essential when you brand this, OK? You’ve got to obviously get it out there. I mean, there’s huge amounts of positivity about DNA testing, OK? And that’s-that’s what you’ve gotta get on – you’ve gotta get onto that uh that-that stepping stone and talk about the positives. Cos, you know, otherwise we’ll be talking about DNA te- you know, how most people think about DNA testing as a threat, not-not a – not a – not an absolutely phenomenal piece of uh science.

Facilitator: [00:41:19] So there’s no point having a poster in the window of the pharmacy saying ‘pharmacogenomic testing’ or even ‘DNA testing’? –

Patient 6: [00:41:24] – No, no, it would take up most of your window.

Facilitator: [00:41:30] Brilliant. OK, well let’s move on to the next one. If you did have a-a pharmacogenomics test, how would you want the results presented to you? Would you want them face-to-face from your pharmacist? Would you want a paper copy? Or would you want an electronic copy? Uh Patient 7, please.

Patient 7: [00:41:53] Uh I believe most probably electronically would be the-the best option because, you know, you know it’s not gonna get lost and it’ll be saved somewhere securely by yourself which you-you can have access to it later on and whenever. Or rather than have been like, you know, face-to-face or even by paper, I would believe – I strongly believe uh electronic would be the best option, myself.

Facilitator: [00:42:14] Thank you Patient 7. Patient 6, please.

Patient 6: [00:42:17] Uh I-I think in the early stages of rollout, it will have to be face-to-face cos you-you need to explain the report. Um you know, most people are gonna just think it’s a piece of paper – what does it mean? So my view, it would, in the – certainly in the fir- early stages, until enough people know what-what it’s all about, it needs to be face-to-face. So – cos I think the public need to be pop- uh educated in what we’re actually doing.

Facilitator: [00:42:40] Alright, thank you. Patient 2, please.

Patient 2: [00:42:45] Yeah, I feel exactly the same. I-I-I think you need to keep that personal link until people are comfortable with the whole meaning of it and how it’s all going to work. So I think face-to-face is really important.

Facilitator: [00:42:58] Right, thank you Patient 2. Yeah, great, OK. Patient 1, please.

Patient 1: [00:43:04] Uh I couldn’t care less whether I get it – get given it or not, if I’m honest. As long as it goes back and that my doctor has it, um you know, I – he’s got all my information about my cholesterol, my blood pressure, my blood type, all that’s – you know, I don’t have it. He’s got it and that’s fine with me. Um I’m assuming it’s not as if they would – I would get – the results would give me any kind of incredibly bad news or anything like that. It’s not like [00:43:34] it’s so – um results of a um an illness, you know, blood test for an illness. It’s just a lit- a set of um my body’s preferences for drugs.

Facilitator: [00:43:43] Yes.

Patient 1: [00:43:44] Um I don’t – I don’t see – I don’t need to have those – carry that around with me – leave it on the doctor’s um – on my notes at the doctors, just like all the other stuff. –

Facilitator: [00:43:55] – As an example, if you were on six to eight – let’s say you’re on six drugs, it might come back that four of them are fine, one of them you need to have a slightly lower dose, so you might reduce from, say, 40mg to 20mg, or four times a day to two times a day. And then the final drug, it may say that your body actually doesn’t respond to that drug at all and an alternative one could be better for you – would be the sort of thing it could show.

Patient 1: [00:44:24] And as far as I’m concerned, that – you know, the-the doctor should keep hold of that, I don’t – shouldn’t have to carry it around in my breast pocket.

Facilitator: [00:44:32] Yes, thank you. Patient 8?

Patient 8: [00:44:35] Um yeah, I-I agree that it wouldn’t bother me going back face-to-face, because if you’ve had it done anyway, they’ll explain what it’s about probably when they do that. So I would be happy with a – just an email or something with the results in. Um so yeah, that wouldn’t bother me. Um but yeah, as for the name of it, uh something friendly, like maybe ‘test and match’ or something, so they know it’s just to match up the medicine or whatever, rather than making it a scientific name. But uh I’ll be happy with an email – I wouldn’t mind.

Facilitator: [00:45:09] Are you in marketing, Patient 8?

Patient 8: [00:45:11] Marketing? Well – well, only so much as the shop [laughs].

Facilitator: [00:45:14] [Laughs]. Brilliant. OK, so that’s how you’d like the result presented. What information would you like the pharmacist to share with you? Patient 8 again.

Patient 8: [00:45:37] Yeah, basically just-just what is – what is relevant. I think you just want to keep the information just short and sharp as to what the actual test was for. I don’t think they need to elaborate cos um, you know, you could get worried or whatever. But yeah, just keep it relevant to what the um – what the medication was. Um yeah, what sort of side effects you might have, possibly, if you took it.

Facilitator: [00:46:03] Thank you. Patient 3?

Patient 3: [00:46:05] Um I-I think th-the interesting point here would-would be if-if you dis-disagree with it – with-with what the pharmacist says, you know. Um there will be people who will automatically ask for a second opinion and, of course, the second op- the second opinion is the G-GP. Um so – so it’s really it’s-it’s-it’s a question of uh also with the – with the cost-cuttings within-within [00:46:35] medicine these days, you know, so the GP wants to give – give you the sort of cheapest but most eff- most effective drug um for your con-con-condition.

Now, if you are therefore prescribed something by the pharmacist, is he sort of doing it on a cost basis or is he actually saying, well actually, you would – you would be much, much better having this particular drug which actually costs £200 a [00:47:05] shot as opposed to the sort of GP who-who would actually say, “Well no-oo, I-I, in fact, wouldn’t prescribe that.” So once again, you come into these boundaries and the balance linked in with cost.

Facilitator: [00:47:17] Yes, thank you. OK, and then what information would you want the pharmacist to share with your GP? Patient 8?

Patient 8: [00:47:34] All of it, even what they haven’t told me, um because I think it’s nice to have that on record, cos then if you go in with a problem, they can look over it and say, “Ah yeah, when you did this, it did bring up such and such.” But um yeah, I would say all of it. Yeah.

Facilitator: [00:47:52] Brilliant, thank you. Patient 4, please.

Patient 4: [00:47:56] You’re saying about sharing with the doctor, surely this information should automatically be going to the surgery and to the pharmacist. And that’s – and then you’re talking this idea of what should they share – surely everything should be in the hands of both.

Facilitator: [00:48:11] Great, thank you. OK. My last question then this evening is then, if Day Lewis Pharmacy were going to set this service up, what advice would you give us? Patient 6, please.

Patient 6: [00:48:51] Yeah, I-I think – I think – I think the – at the core at the moment is, certainly after COVID and everything else, depending on how quickly you’re gonna bring this in, don’t you lose that personal contact with the customer. I-I-I think it’s-it’s vitally important for communities. I mean, I-I think that, actually, if you look at um what we’ve lost as communities over the last – what, ten years – 10, 12, 15 years, most-most places have lost their local pub, they’ve lost the Post Office, OK? [00:49:21] It’s absolutely vital um that um the bedrocks of our communities, i.e., the doctors and the pharmacists, stay where they are.

OK, technology – technology absolutely can uh enhance their service, but we-we all need human contact, and I think that’s becoming very [laughs] – I mean, it’s actually becoming very evident, I think, in-in how many antidepressants I was reading have been dished out during the [00:49:51] COVID crisis. So-so fundamentally, if you – if you want to uh, you know, carry on um – carry on as a business and a business that stands on its [legs? 00:50:00], is, don’t lose that personal contact. It’s vital.

Facilitator: [00:50:05] Great. Thank you Patient 6. So the question is, if Day Lewis was going to set this service up, what advice would you give us? I’ll go to Patient 2, then I’ll go to Patient 8.

Patient 2: [00:50:15] Uh I-I feel exactly the same and-and Patient 3 was just agreeing too, it-it’s to do with the personal touch, it’s to do with relationship. Uh we-we all need that and-and certainly if you need medication, you need that reassurance. Um so I think if you stick to what they’re certainly doing here, um and-and giving that um personal touch, then that would be fine. Just what we need.

Facilitator: [00:50:42] Thank you Patient 2. So what adv-advice would you give us, Patient 8, please?

Patient 8: [00:50:46] Um in my opinion, um because it is uh-uh a new idea, I would like um some sort of leaflet or letter from my doctors surgery, introducing the service um and saying, you know, that it’s available at the local Day Lewis pharmacy. Because if it came from anybody else, I would be worried that it was a bit of a scam or something, so I would actually want it to be introduced to me by my doctors surgery.

Facilitator: [00:51:15] So you’d like the GP and the pharmacist to work together – or the-the practice and the pharmacist to work together?

Patient 8: [00:51:20] Yeah, but I would want something from my doctor so that I would know it was a genuine service that’s being offered.

Facilitator: [00:51:29] Thank you. Any more advice from – for us? Is that Patient 1 saying yes or … ? Any more advice, Patient 1?

Facilitator 2: [00:51:51] Patient 7 has his hand up, Facilitator.

Facilitator: [00:51:53] Yeah, OK.

Patient 7: [00:51:55] Yeah, I was gonna ask you, how are you gonna [push? 00:51:57] um a patient that you are doing the service? How are you gonna promote it? I mean, at least about to start it, but how are you gonna promote the message to your – to the patients?

Facilitator: [00:52:07] So how would you recommend we promoted it?

Patient 7: [00:52:11] I dunno, it’s like not everyone goes to their surgeries, do they? I mean, the pharmacies. So unless they go there, you know, or like um kind of what the lady’s mentioned, if you hear it from your GP, that’s beneficial, that’s secure as well. But at the same time, what if someone don’t – pharmacies every so often, how would you notify them? So I don’t know if there’s another option. –

Facilitator: [00:52:35] – That’s a very good question. Thank you, Patient 7. Thank you. OK, has anyone got any further comments or questions before I wrap up? No? Fantastic. OK, so what’s gonna happen next is, in a minute, Facilitator 2’s going to stop recording and then what we’ll do is, we will then um have this recording transcribed, so everything you’ve said will be converted into Patient 1 for [name], Patient 2 for [name] and so on. So it will all be [00:53:05] completely anonymised in the next uh couple of days and then we’ll make sure that this recording is completely deleted, so-so-so talking about privacy earlier – really important. So everything will be completely anonymised.

Um then um I’m going to review all the data from the GPs and from the pharmacists and from tonight, and then in May I’m going have another focus group bringing back together three GPs, three pharmacists and, hopefully, three of you, [00:53:35] so we can um bring it all together. And then have all the pharmacists, the GPs and the-the patients on one call to share back the results to start developing some of the training materials and some of the information. We thought it was best to segregate um the GPs and the pharmacists initially, um and I think tonight has been a really great diversity. So I’m so pleased with the-the feedback we’ve had tonight.

**Pharmacist focus group transcript**

Pharmacist-Focus–Group–Novel–Pharmacy–Service–Design–20201215

Facilitator: [00:00:04] The purpose of this focus group, as you know, is to develop a novel pharmaceutical service, but we haven’t really said what that is because we want to get your first ideas about it. So, I’m going to share some background details with you on an innovative area of healthcare, starting with a short briefing on personalised medicine, um, followed by the main reason for this focus group which is about pharmacogenomics testing. [00:00:34] OK? So, that’s the, that’s the thing we’re going to be looking at. The personalised medicine is the combined use of genetic, environmental, lifestyle, clinical and other unique patient factors to influence prescribing.

We know that some medicines don’t work because our body is not designed to deal with them in the way that we expect. Sometimes our liver will clear them away too quickly and in others they won’t clear them at all, meaning that we’re more likely to have side effects. We now have the technology [00:01:04] which allows us to look at patients’ genes and decide in advance what the best drug or dose is for them. With the advent of lower cost DNA testing this new approach will soon be available for everyone prescribed a medicine for which a genomic test is known to be able to improve prescriber decision making. Community pharmacies could, in the very near future, offer this service. Um, I’ve lost my [unintelligible 00:01:31].

This focus group is designed to get your views on [00:01:34] the technology and idea of pharmacists offering a pharmacogenomics test to patients when they start a new medicine. So, I don’t know if this is something you’re familiar with, uh, as a, as an approach, but pharmacogenomics uses genomic information to predict an individual’s response to drugs and can deliver personalised medicine rather than the traditional one size fits all. Pharmacogenomics derived from the words for pharmacology, the study of drugs, and genomics, the study of genes. [00:02:04] Patients metabolise and eliminate drugs at different rates, resulting in some patients requiring a lower dose, some a higher dose and others requiring a different medicine altogether. And this all contributes to about 30 to 50% of patients not taking the medicine as intended for long term conditions, often resulting in unnecessary hospital admissions.

And by 2025 the new NHS Genomics Medicine Service will be integrated into routine healthcare. Pharmacists will be an integral part of this [00:02:34] service from explaining test results to patients to alerting prescribers on significant gene/drug pair interactions. To date, pharmacogenomics testings has been piloted in a range of settings, including GP surgeries, hospitals and community pharmacies, um, but that’s in other parts of the world, not currently yet in the UK. So, um, I wanted to show you, um, a flowchart of what, uh, [00:03:04] such a service offer might look like, um, and then to show what a report might look like. And then I’m going to ask you some questions, OK? So, I’m just going to share now, uh, if I can get the right thing to share. This one. Right. Can you all see that?

Pharmacist 3: [00:03:30] Yes.

Pharmacist 8: [00:03:31] Yes.

Pharmacist 5: [00:03:31] Yes.

Facilitator: [00:03:32] Is it big enough to read?

Pharmacist 3: [00:03:33] Yes.

Pharmacist 8: [00:03:34] Yes.

Pharmacist 5: [00:03:35] Yes.

Facilitator: [00:03:36] OK. So, I’ll just talk you through the boxes just in case it’s not quite big enough. So, the idea is that the patient visits the pharmacy for a medicines review, um, and at that point the patient could be offered a pharmacogenomic test. Um, so the test is taken, um, and then the test results indicating potential gene medication, um, link or interaction is returned in ten days. At that point the pharmacist discusses the results with the patient and [00:04:06] then there are some options.

You could, um, either then discuss it with the patient and then refer to the prescriber for some changes to prescription or you could decide that no change is necessary. If you ref-, if you refer then to the prescriber for a change in prescription then they would either accept the change or reject it. So, that’s, that’s what that pathway could look like, and, um, the sorts of reports that these, um, uh, [00:04:36] companies who produce the tests produce look like this. Can you see a report now?

Pharmacist 3: [00:04:43] No, still got the flowchart.

Pharmacist 5: [00:04:43] No.

N4: [00:04:44] No.

Facilitator: [00:04:45] OK. Then I shall stop sharing and start sharing again. Uh, pick the right document. Does that look like a report?

Pharmacist 5: [00:04:59] Yes.

Pharmacist 3: [00:04:59] Yeah.

Facilitator: [00:05:00] OK. So, you can see it says, ‘Personalised Medication Report for Test Patient.’ So, this is a made up one. Um, there’s information about the current medications and it’s kind of colour coded. So, a red dot means a major prescribing consideration, yellow a minor, uh, and green is sort of business as usual. And the sorts of recommendations are, um – so if you see here for codeine or paracetamol, there’s a significant result [00:05:30] that might require altering this medication.

So, the idea is that as a pharmacist you’d get this response, this report, uh, and you can sit down and discuss it with the patient. So, as well as, um, medicinal information, medicines, uh, information, there might be some genetic, some general genetic test information, and there might also be a, uh, uh, a recommendation. So, here’s a strong recommendation to avoid [00:06:00] codeine use due to lack of efficacy. OK, I’m going to stop sharing that for now, but we can come back to those if you would like to see them, uh, at any point.

So, you might want a moment just to, uh, mull that over. Uh, and so I want to ask my first question now which is, having seen this what would be your first thoughts on this new technology, on using pharmacogenomics and using that information [00:06:30] in this way?

Pharmacist 3: [00:06:33] My first thought is, my grandmother recently had to start taking losartan. Previously she had taken ramipril, but it had caused her kidneys to deteriorate. So, if they had known beforehand, the kidney function wouldn’t have gone down at all because they would have known, “OK, don’t use the ramipril, go for the losartan instead,” immediately stopping her – she’s 92, she doesn’t – [00:07:03] she gets confused anyway. Suddenly having the doctor switch medicines on her doesn’t help.

Facilitator: [00:07:11] Yes. OK. Um, any other thoughts about the use of this technology?

Pharmacist 5: [00:07:16] I think it’s good. I think it’s futuristic. I think it’s innovative. I think it will really help patients, um, not only identify what they should and shouldn’t be taking, but also provide a more individual approach to what they are taking, because as we know, one size doesn’t fit all. I think a lot of medicines are prescribed on a trial and error basis. So, they go away with the medicine with a low percentage that’ll actually work, but just with kind of evidence on different kinds of people. So, if we can get the information from technology that’s so, [00:07:46] it’s so individual, I think it’s a fan- – the first thought I had was, “That is an absolutely fantastic idea. It’s going to save both the patient and the NHS a lot of – well, a lot of money and also a lot of care. So, yeah, I think it’s great.

Pharmacist 1: [00:08:00] Yes, I think it can also reduce like hospital admissions and things like that in terms of side effects and how the medication reacts with the specific person’s body and things like that. So, yeah, a great, um, innovation I think.

Male 1: [00:08:19] It think it’s –

Male 2: [00:08:20] [Unintelligible 00:08:20] – uh, sorry. No, no [unintelligible 00:08:23].

Male 1: [00:08:24] Yeah, you go ahead.

Male 2: [00:08:26] And definitely for adherence as well. It will support a lot of the adherence. It will make our [unintelligible 00:08:31] much easier to, to deal with. So, I think it’s a, I think it’s a very, very great idea to be honest. I think – yeah, it depends on the – now, it depends on the cost as well. There is a lot – the, the idea is good, but it has to be organised quite well because you have to have a good communication with the GP as well to make [unintelligible 00:08:54] because, for example, if it takes ten days to get the result, but for example the GP want to start the medication and then expect like a blood test in ten days to see how the body’s responding, you have to synchronise all these new things. So, yeah, but the idea, the, the, the idea is very good.

Facilitator: [00:09:19] [Unintelligible 00:09:19]?

Pharmacist 7: [00:09:19] I think it’s a good way forward –

Male 1: [00:09:20] Uh, it’s also really good –

Facilitator: [00:09:21] Pharmacist 7, sorry, you were –

Pharmacist 7: [00:09:23] No, that’s OK. I think it’s a good way forward, and I think it will get patients involved in their care a bit more. So, if they’re able to like see it on paper and – you know, in community pharmacy we’re at the forefront, so we’re able to – we’re in the community, we’re accessible, we’re going to be able to like, once we have enough training, um, to just be there to explain it to them. So, do think it’s a good service.

Facilitator: [00:09:46] Sorry Pharmacist 8, I think I stopped you then.

Pharmacist 8: [00:09:51] Uh, well, no I hadn’t started, but, um, I think, not that it’s a terrible idea, but this – just to recap for someone who’s worked, um, had a career in the pharmaceutical industry I, I think this idea is naïve. Um, I spent a long time working with companies, pharmaceutical companies whose sole objective [00:10:21] is to have prescribers prescribe their products. If anybody thinks that a pharmacist in an, in an ad hoc test is going to potentially reduce the amount of prescribing of a pharmaceutical company’s drug by doing a test on their patient downstream of the prescriber, they’re very much mistaken. That will never happen.

Pharmacists have never been involved really [00:10:51] in the decision-making process as far as pharmaceutical companies are concerned which is why we very rarely see them. And whatever decisions are made based on pharmacogenetics will not be left in the hands of a group of people, pharmacists, with whom the pharmaceutical company does not have a well-developed relationship. It will be moved upstream, and the decision will be made upstream based on the, the tests.

[00:11:21] It will not be – a GP will not prescribe a medication and then the pharmaceutical company will take the risk that some pharmacist in a high street will feedback to the, the GP that this is the wrong medication That will never happen. Indeed, it doesn’t happen. And the pharmaceutical company, trust me, who, you know, spend a larger amount of money on marketing than they do on R&D – it will never, it will never happen. It’s a nice idea, it’s a theoretical idea, but [00:11:51] I think you underestimate the degree to which pharmaceutical companies influence prescribing and the prescribing landscape.

Facilitator: [00:11:59] OK.

Pharmacist 8: [00:11:59] And to come up with an idea, an ad hoc idea which might dovetail into a community pharmacy service is nice, but is to completely underestimate not only the future of retail pharmacy but what pharmaceutical companies will do to stop that happening, and I –

Facilitator: [00:12:16] OK.

Pharmacist 8: [00:12:16] – and that’s, and that’s it.

Facilitator: [00:12:18] That, that’s great. Thank you for that comment. Now

Pharmacist 2: [00:12:20] Can I say something?

Facilitator: [00:12:20] – um, Pharmacist 9 you had your hand up I think?

Pharmacist 2: [00:12:23] Can I say something?

Pharmacist 2: [00:12:26] Um, yeah, I mean I do agree with Pharmacist 8 that, um, it’s almost at the wrong end, the, the testing’s at the wrong end of, of the process and that any – the testing would be more, more beneficial earlier on and the, the NICE guidelines for basically every care category, you know every healthcare category in the BNF, the whole lot would all need to drastically change. And for us to sell a service or even [00:12:56] at some point that service be commissioned to them, (sighs) it makes things very complicated, I suppose is what, is my immediate thought. Um, you know, from the landscape the way it is now to introducing the benefits of this individualised care, I think it’s, it’s a, a long time in, in the making.

Facilitator: [00:13:18] Thank you, and we’re going to pick up on some of these things with the next question. So, um, Pharmacist 10 I think you put your hand up?

Pharmacist 10: [00:13:27] Um, I’m just, um – I’ve got one question on who’s going to pay for the test? Is it, um – uh, is it paid by the NHS or by the patient? Um, I mean, we could, we could try it to see if, um, if it, um, if it works in, in the market. If it, if it fails, it fails. I mean, um, it’s better that we’ve, we’ve tried, and it failed than, uh, not to try and, um, have this thought, [00:13:57] “Well, what if we tried?” That’s, that’s my opinion, but the cost for me is, is quite important. Who’s going to pay for the test?

Facilitator: [00:14:07] Yes. It’s, it’s a good question. Um, um, I, I think, um, there’ve been conversations around private services, but, um, there might – you know, I don’t want to, uh, lead on that. So, um, has, has everybody said something about their first thoughts? Are there any other comments?

Female: [00:14:30] I think Pharmacist 4 and Pharmacist 2 still have their hands up.

Pharmacist 4: [00:14:31] Um, I think because of –

Pharmacist 2: [00:14:33] Yes. I would like to say, you know, yes, it is true that the pharmaceutical industry has got a lot of power, but at the same time I think if they patient is experiencing the side effect, yes, and we are able to explain to the prescriber where this side effect comes from, I think that the prescriber will think twice before not changing the medication, because, [00:15:03] yeah – I, I understand if the patient doesn’t complain and everything is fine, but when the patient starts complaining, yes, the pharmaceutical industry I don’t think is so powerful anymore, I think.

Facilitator: [00:15:19] OK. Thank you. Pharmacist 4?

Pharmacist 4: [00:15:24] Um, I was going to speak about, um, some of these guidelines that the NICE presents, most of them are [line breaks up 00:15:29]. So, if it helps us in terms of, um, catering individualised therapy, like for example if you look at diabetes care, after metformin it’s a bit of a grey area. We don’t really know, uh, individuals are different in terms of their genetic makeup and their physiology. We don’t really understand what’s good, we just do one size fits all at the moment.

If we had, uh, some kind of specialised service [00:15:54] where we could determine whether one individual might benefit from a, uh, like, like, uh, DPP-4 inhibitors or another therapy, it paves the way for providing that, uh, specialised therapy that’s suited for the individual’s needs. And, and in the long term, not only will it help with polypharmacy, but it will help with, um, hospitalisation and also reducing the cost to the NHS. But it could potentially [00:16:24] also be a really good service privately, uh, for the, for the business side of the pharmacy as well. But I, I can also see the challenges ahead as, uh, Pharmacist 9 and, uh, Pharmacist 8 pointed out. So, uh, we’ll see what the pilot says as well. So …

Pharmacist 8: [00:16:40] As someone who’s sat on, um, global meetings of companies like Novartis and Roche, and you cannot – unless you’ve sat there and listened to the discussions, these people will go through the market performance by country by country and go into very, very granular detail as to why a product is not being prescribed more in Germany than in France, than in Italy, [00:17:10] than in Moldova, than in Ukraine. The medical directors of each country volunteer information as to why that might be. Who are the inspirances? Who are the key opinion leaders? What Glaxo is going. What AstraZeneca. Anything.

Anything whatsoever that comes up that says, “Losartan is not being prescribed in the UK. We think it’s because pharmacists have decided to do pharmacogenomic testing,” will be the focus of any [00:17:40] amount of millions that they will require to reduce any loss in their product. And if that means changing the side effect profile, changing the drug or whatever, they will skirt the problem. There is no way that a pharmaceutical company will let a pharmacist influence an upstream prescriber, and they will make sure that doesn’t happen. They’ve being doing it for years. So, you have to think, you know, the genomic testing is a nice idea, but it’s not going to be – the companies will not let [00:18:10] pharmacists do it –

Facilitator: [00:18:12] Mm.

Pharmacist 8: [00:18:12] – decide. It, it, it won’t happen. There’s – not in a million years.

Facilitator: [00:18:16] OK. Well – um, no that’s –

Female: [00:18:17] Pharmacist 8?

Pharmacist 8: [00:18:18] Yes.

Pharmacist 5: [00:18:18] I’m sorry –

Female: [00:18:19] [Unintelligible 00:18:19] –

Facilitator: [00:18:20] Sorry, Pharmacist 4 [unintelligible 00:18:20] who – Pharmacist 11, were you wanting to reply? Sorry, I – Pharmacist 4’s still got his hand up. If you could put it down for me.

Pharmacist 5: [00:18:27] It’s OK. I mean, like haven’t we all – the, the whole purpose of this group meeting is service design, and even if it’s theoretical – and pharmacists are not living like we did like 40 years ago, it’s changing more rapidly than ever. So, even if it’s a slim chance and even if they do have to pay privately, which they are doing in other countries, they are doing it in America, they’re doing it in Australia, if the patient wants to know individually their own risk factors, their own gene code and to try [00:18:57] and talk to the prescriber themselves and make a decision, then we should be able to facilitate that. I don’t see any problem with designing a service. Even if it’s totally unrealistic to some people, there is a chance that it could be marketed privately, even though the [unintelligible 00:19:13] might say, “Uh, no we’re not – ”

Pharmacist 8: [00:19:16] I, I agree with you, except the different is of doing it privately, it’s like you can do a lot of stuff privately but what we’re talking about here, in order to be commissioned going back to the point of what is the value of a commissioned service, is to bring, bring benefits to the patient, then you have to change something. And it means that you’re going to have to talk to the prescriber and put a case to them to say, “I’ve done the genomics test on your patient, and you’ve prescribed the wrong thing.” Now, think of the relationship [00:19:46] between the patient and the GP. You know, that’s a very fraught area. You can’t ring up a GP and say, “You’ve given effectively a medication to someone that’s causing them side effects or is inappropriate.” That’s a very difficult discussion you’re going to have.

Facilitator: [00:20:01] Well, I think the idea is that that would be some kind of, um, shared decision making with the patient. Uh, Pharmacist 3 you had, uh, your hand up?

Pharmacist 3: [00:20:09] Pharmacist 8?

Pharmacist 8: [00:20:09] Yes.

Pharmacist 5: [00:20:09] There’s two issues that I see with what you’re saying.

Pharmacist 8: [00:20:13] OK.

Pharmacist 5: [00:20:13] One, you’re assuming that everybody would have a very similar genomic profile and therefore one drug would not work for the whole country. It’s unlikely that it would be like that. It could be, we’re saying one drug for one patient, yes. Another drug for another patient, no. It’d make a very small amount of difference to the big pharma companies. Also, we already tell GPs that they’ve made mistakes when it comes to prescriptions.

The NMS service, we tell them, [00:20:43] you know, “This patient can’t use that inhaler. They don’t have the suction power. They can’t use it.” We tell them, “They can’t take that medication with the metformin. They’re suffering from diarrhoea. You either need to make it modified release or go somewhere else.” We’re already interacting with the, with the prescribers. We’re already influencing medications that are being prescribed to our patients. I have doctors phone up and say, “OK, you’re telling me this drug is unavailable. [00:21:13] What else can I give?” We’re already part of that communication. We are already part of that process. This is –

Pharmacist 8: [00:21:20] And do you get paid for that?

Pharmacist 3: [00:21:20] – just a step up.

Pharmacist 8: [00:21:22] Do you get paid for it though?

Pharmacist 3: [00:21:22] We are accessing more information that the GP did not have.

Pharmacist 2: [00:21:26] [Unintelligible 00:21:26].

Pharmacist 3: [00:21:27] What we do is, we tell the patient, “This is the way it has always been, but your genomic profile shows that this is actually not the best thing for you. The doctor didn’t have that information available. He went, or she, went with what they have always been told to do by the NHS. We’ve looked into it a bit more. This actually would work better for you.” It’s more about a liaison, more information feeding back. GPs love more information. I don’t see any issues with this at all.

Facilitator: [00:21:58] Um, can I ask some, um, more questions to move the, the, um, the view on? Uh, so if we could think for a moment about your patients. So, what do you think will happen for your patient if you con- – if you were to conduct, uh, pharmacogenomic testing? What do you think, um, their reaction might be?

Pharmacist 8: [00:22:22] Well, it’s very obvious. If you test someone and say, “You’ve been given metformin and you should have been given something else,” you have to manage a whole number of scenarios. But not least the fact that they’ll ask you why and you’ll say, “Well, I’ve done a genomic test on you,” (chuckles) and they’ll say, “OK, well why didn’t my GP do that?” and he says, “Uh, he’s not allowed to.” I’ve no idea – I mean, let’s assume he hasn’t.

Based on the scenario that Pharmacist 3 is putting forward it, it, this is, you know, this is disruptive. [00:22:52] This is disruptive. I mean, you are moving – and – it’s, it’s a step too far for pharmacists. I don’t mind having this within a suite where you have a research focussed organisation like pharmacy, but to move from MURs, NMSs to a de facto objective test, not a clinical suggestion that this person is on the wrong medication, is something, is something much [00:23:22] more and it needs very careful managing. And it can’t be done in, in an ad hoc way in a consultation room –

Facilitator: [00:23:27] Yes –

Pharmacist 8: [00:23:28] – based on a, you know, a blood test.

Facilitator: [00:23:30] And I think that’s really helpful because, um, uh, as Pharmacist 11 was saying there’s an opportunity for us to think about how we move that from an ad hoc, um, conversation to a planned, uh, service that we might be able to offer, uh, in a shared way, through shared decision making with patients, and then drawing in and involving their, uh, GPs afterwards. So, I don’t think this is, um – the intention behind this where it’s been done in other countries isn’t to standoff patients against their GPs. That isn’t how it’s manifested itself. [00:24:00] So, if we come back to thinking about what the patients might, might think or get from this, um, any other views from – ? Pharmacist 7?

Pharmacist 7: [00:24:08] Hi. Um, so I think it could go two ways. So, there’s patients that generally want to, um, obviously be engaged with their care. So, those ones will probably be really interested. And then there’s the other side of patients that, you know, they just want to listen to what their GPs kind of have to say and they’re not really like proactive. So, there’ll, there’ll be both ways if the service was to go forward.

Pharmacist 8: [00:24:34] I just have one –

Facilitator: [00:24:34] Uh, Pharmacist 3 did you – ? Sorry, Pharmacist 3 you had your hand up I think?

Pharmacist 3: [00:24:39] Yes. The, the way I see it is, I have a number of patients who I have done NMSs on, one NMS a month for three months, because they’ve been trying out their medication and it didn’t work, it didn’t work, it didn’t work. The idea that they could skip all those intermediate steps and go from one that – basically just go to one that does work. They would be so relieved because people don’t like being messed about. They don’t like trying everything out all the time [00:25:09]. If you’ve got an option to jump straight to the right answer, they’re going to be keen on it.

Facilitator: [00:25:16] Uh, Pharmacist 9 did you have a comment? Your hand is up.

Pharmacist 9: [00:25:20] Yeah, I, I think the, the biggest challenge, and Pharmacist 8 and Pharmacist 3 have both touched on it, is, um, managing patient expectations with the results in front of them, and then teaming up with the patient, the pharmacist and the GP to find the next best step that suits the NHS care pathway and also the NHS budget that they’re willing to spend. Within private care they can l-leap straight to the answer as Pharmacist 3’s kind of indicated, that the, the, the testing may [00:25:50] point towards, but whether the NHS can pay that next step is, is a, is a whole separate debate. And so managing the expectations is, I think, and the collaboration between patient and GP and the, and the results and us as a facilitator is, is kind of my view on, on all of that.

Facilitator: [00:26:11] Pharmacist 4 did you have a comment?

Pharmacist 4: [00:26:13] Yeah, I was just going to follow up on what Pharmacist 3 and, uh, Pharmacist 9 [line breaks up 00:26:16], uh, I think it’s a step ah- – it’s a steppingstone towards this collaborative approach that the NHS is pushing towards such as the PCN and, um, and, and what we need to remember is if this is going to [line breaks up 00:26:30] GPs’ time, I think it’s going to be helpful in that sense as well. And it’s, again, a kind of a shortcut as to finding out what, what the patient’s needs are and patients are the biggest drivers for the NHS items at the moment [00:26:42], and it’s not the – I don’t – it’s not the pharmaceutical companies. So, that’s the most important thing you need to remember. If it, if it caters a patient centred approach and we, we’re going to put the service and, and the patients are satisfied with it, I think it’s going to be a real positive for the future of pharmacy.

Pharmacist 8: [00:27:02] Pharmacist 4, I agree with you, it’s patient centred. I’m just wondering at what level that level of care, whether the GPs will jump into that space. The other thing I would be interested to, to ask Facilitator whether she has information, or anybody, of what percentage of patie- – of medicines currently prescribed benefit from genomic testing? That is, what would be the, the significance of changing one person to another product? Inhalers, uh, co-codamol, painkillers, do we know what percentage of prescriptions would be affected?

Facilitator: [00:27:37] I don’t know the answer to that.

Pharmacist 8: [00:27:40] I mean, if you think of all the drugs that we prescribe, inhalers, largely antihypertensive drugs, um, antidepressants, painkillers, in fact there’s only a very small proportion of those which when a genomics test is done are likely to modify the, um, the prescribing. So, whether the business is itself scalable is questionable.

Pharmacist 3: [00:28:07] There’s also the other aspect. Um, I have a lot of asthma in my family, and I know that there’s about 10% of asthmatics who can’t use ibuprofen. To that extent, I recommend that anyone that uses, that has an inhaler doesn’t use ibuprofen. I would imagine that this test would reveal those that could and those that couldn’t, which would then leave more options for pain relief for those patients who had always had, “Ibuprofen, “no, do not touch.”

Facilitator: [00:28:39] Um, so, um, if we think – sorry, I think Pharmacist 11 has something to say.

Pharmacist 5: [00:28:45] Yeah. I was just going to say, I mean, if theoretically it was to go ahead, I don’t think we should start with genomic testing in general and do everything. We should start with something that’s small, something that’s precise, something that’s easily identified as a problem. Something that say – I can’t even think of a genome off the top of my head, but people with this are regularly getting mistreated, and then we can try and identify the problem, quantify the benefits to the patient, to the doctors, and you know try and start with a real focus [00:29:15] on something that’s small.

Um, I mean with regards to provision of the service, I do think maybe when Pharmacist 8 said it’s doubtful, it depends on what kind of pharmacy you’re in. Like the pharmacy I’m in at the moment doesn’t do – it does mid-range items. We have an excellent skin clinic. We have weight loss. We have botox. We have all sorts of services that are running constantly. So, I don’t spend all my day checking, I’m always busy doing injections and things and PGDs. Um, but in the previous branch [unintelligible 00:29:44] (significant background noise) –

Facilitator: [00:29:50] I can hear some background chat. I don’t know if anyone’s got their radio on or something? Anyway, um, can I ask, um, now about how pharmacogenomic testing might affect your approach to medicines optimisation? This is more about you personally, um, and how this might affect you and your role as a professional [unintelligible 00:30:15]? (Significant background noise).

Pharmacist 4: [00:30:18] There’s background noise going on.

Facilitator: [00:30:20] Is there another ..? (Significant background noise). There’s, there’s another, um, chat going on. You know, perhaps if we all mute – if you all mute for a moment we can see whether it’s any one of us. OK, right. [00:30:50] Um, so have a think about this question, about, um, whether pharmacogenomic testing, how – what affect it would have on you and the service you provide, um, and, um, whether it affects your professionalism, the impact it might have there. (Significant background noise). Any thoughts? You can unmute yourself (chuckles) or put your hand up if you have a thought about it.

Pharmacist 4: [00:31:26] Could you repeat the question again please?

Facilitator: [00:31:28] Uh, yes, of course. Um, it’s, um, how could pharmacogenomic testing affect your approach to medicines optimisation? Do you think it would have a role, um, or could it affect your professional standards? Do you think that offering this other service might put you in any difficulty? (Significant background noise).

Pharmacist 3: [00:31:55] I don’t think it would conflict (significant background noise) anything at the moment. It’s about getting the best – I don’t think that it would conflict with anything at the moment, because it would get – it’s about getting the best out of the medicines for the patient. If a patient needs to take three medicines to control their blood pressure, but it turns out that two of them don’t work for that patient at all because of their genetics, reducing them to one, benefit to the NHS, benefit to the patient.

[00:32:25] It would have to be done quite carefully though, because you don’t want to, uh, l-launch into the idea of, “Well, this is how we’ve always done it, but we’re changing it now.” S–some people have always followed, “The doctor is God, must obey the doctor.” It’s not so much in the younger generation, but the younger generation are less likely to have polypharmacy. So, it’s (sighs) – I feel it would help [00:32:55] with medicines optimisation, but I also feel that it’s going to, it’s going to cause a bit of disruption with some of the older patients, because there was like, “Well, that’s not that the doctor said. I’m going to go with the doctor.”

Facilitator: [00:33:11] But do you think that, um, that picks up Pharmacist 8’s point of earlier about, um, managing the relationship carefully with, with the GPs?

Pharmacist 3: [00:33:20] It’s more about the – I feel it’s more about the patient because I’ve, I’ve had patients walk in and say, “The doctor told me to get this,” and you’re looking at them and going, “No, that’s really not the best idea for you,” but the doctor told them and they’re going to stick with what the doctor said. I normally find with my patients that is normally the – those in the over 70s, over 80s category.

Facilitator: [00:33:46] OK.

Pharmacist 3: [00:33:46] They came from an era where doctor was God, you do what the doctor says.

Facilitator: [00:33:50] (Chuckles) Um, so, uh, Pharmacist 11 did you have a follow up?

Pharmacist 5: [00:33:55] Um, yeah. I mean, you mentioned medicines optimisation, and, um, the basics of that is something that’s clinically effective and cost effective. Um, the only problem with this service would be that it’s probably not cost effective at all. It would have to be someone that is targeted and is definitely going to be switched to save money. So, that creates an imbalance in the cost-effective side of it, but, I mean, otherwise you just incorporate it into what you say in your consultations as like, um, a clinician looking at all the medicines, are they appropriate, the patient’s clinical profile, presentation of symptoms, [00:34:25] things like that. Um, you would build it into your own, um, – yeah, you’d just build it into your own practice really. I don’t see how it would affect it negatively, other than the cost.

Facilitator: [00:34:36] Pharmacist 2 did you have a point?

Pharmacist 2: [00:34:39] Yeah. I think as well that, you know, at the beginning until we don’t, we don’t gain confidence with the service, maybe to be successful the patient should come to ask, because he’s complaining about any side effect. Then, using the service we can experience and then when we see other different scenar-, similar scenario in other patient, we can approach the patient proactively, but at the beginning to avoid any, any risk I would wait. And of course you advertise the, the service [unintelligible 00:35:17] to wait until the, the patients [00:35:19] approach us, and then it should be better.

Facilitator: [00:35:24] Um, thank you. Pharmacist 4 did you have something?

Pharmacist 4: [00:35:28] Yes –

Facilitator: [00:35:28] I’m, I’m seeing your hands are still up, so, um, if you’ve made your point –

Pharmacist 4: [00:35:29] – I was going to mention another point.

Facilitator: [00:35:31] Yes.

Pharmacist 4: [00:35:32] Yeah. Uh, can you hear me?

Facilitator: [00:35:35] Yes.

Pharmacist 4: [00:35:36] I was just going to mention about the, uh, um – as pharmacy we are very accessible for the patients, and, and most of the time patients come in and they say, “OK, this treatment that was given to me is not working.” And normally it’s the pharmacy that they, they try to contact unless a doctor speaks to them to follow up. So, in that sense, uh, it’s, it’s, um, we have to remember that it’s – it will help us in, in terms of dealing with that kind of scenario, and, uh, yeah, [00:36:06] that’s something quite important we have to bear in mind.

Facilitator: [00:36:11] Any other points about this question? Uh, Pharmacist 8 you’re on mute. You have to unmute yourself. I don’t know if Facilitator 2can un- … can you unmute yourself Pharmacist 8?

Pharmacist 8: [00:36:31] Yes.

Facilitator: [00:36:32] (Chuckles).

Pharmacist 8: [00:36:32] Um, pharmacogenomics testing is very often not done to swap drugs completely, but helps with starting dose, as mentioned previously, to try to reduce side effects particularly, for example, cytochrome e450 antipsychotic drugs. So, it doesn’t mean that the drugs will not be prescribed but maybe the starting dose would be different. And once the pharmacy [unintelligible 00:37:00] has realised that there’s a different starting dose, [00:37:02] as happened with [sl. rosuvastatin 00:37:03]. Many of you may remember that rosuvastatin originally came out at 40mg. That was too aggressive. It’s now available at 5mg because the stating dose was too high. The side effects of muscle spasms.

So, it’s not a complete swap but really a change in starting dose, modifying perhaps even, um, you know certain prescribing frequency, and that’s likely to be a decision, particularly if it’s initiated by the GP, if it’s already been started. And if it foun- – if it’s found, as happened [00:37:32] in, you know, Australia that the main benefit is in initiating starting doses in statins, warfarin and so on, then the decision will be made by the prescriber. It’s not a complete switch. This is not going to be used.

Pharmacogenomics has not been used generally to suddenly – to say, “Do this, not that,” but rather maybe fine tune various things because of the idiosyncratic nature of individuals. Even though you may have a, a particular genomic proclivity [00:38:02] for one drug, it does not mean that you have a zero response. You may just have a better or less response. So, so, we’re talking about fine tuning here, you know, first of all.

Facilitator: [00:38:13] Um, Pharmacist 7 did you have a point you wanted to make? No. Pharmacist 9?

Pharmacist 9: [00:38:18] Um, yeah. I think it’s, it’s potentially a tool to use to optimise meds, and it, it’s, it’s just some-something else for the clinicians to help them make the decisions, whether it’s starting doses or choice or appropriate medication. I think it’s – you know, it’s, it’s an extra tool, and if people are willing to pay for that privately, then that’s, that’s great. If at some point in the future the NHS deems it cost effective, then, you know, in certain, [00:38:48] certain specific areas, then that’s great. So, to me, it’s, it’s a, it’s an extra tool.

Facilitator: [00:38:54] Thank you. Pharmacist 10?

Pharmacist 10: [00:39:00] I see, I see the testing, um, not in the starting point or where at least the pharmacy can intervene, but, uh, sort of in alongside the treatment or, um, um, in, in, in the middle of the treatment where something doesn’t work, the patient gets more and more medication, more painkillers, more co-codamol. Um, probably everybody’s seen co-codamol is prescribed, um, like, like [00:39:30] nothing, as Smarties if, uh, if I’m explaining myself properly.

Um, so I think probably for some people or the pharmacy can come with this test (significant background noise) when patients get, get frustrated that their treatment is either not working or the doctor prescribed, uh, prescribes more and more. This is, this is where I see it, and it, it can, um – if indeed the test comes back, um, it can [00:40:00] explain a lot of things, and it can, uh, help the GP, uh, turn the treatment around and, um, it, it could save time, patient’s, patient’s time and frustration and the GP’s time. That’s where, that’s where I see it.

Facilitator: [00:40:17] Um, I’m mindful of the time. So, I would like to, to move on and ask you, um, now what you think about your concerns and your challenges in delivering a pharmacogenomic service within your community pharmacy? Uh, and so I’m thinking, um, any factors that you think might be, might be concerns or challenges, um, you know, small scale, large scale? Pharmacist 3?

Pharmacist 3: [00:40:46] The way I see it is, as mentioned before, the cost of the test, although I have been looking. Some tests – some DNA testing can be as low as £10.00, although that is just for paternity, all the way up to £140.00 something. So, we’re looking at a big range of prices here. Secondly, reassuring the patients about the confidentiality of the reports, because things like ancestries [00:41:16] DNA tests and such like, everybody knows they’re put out there so that you can find genetic links. So, people quite often might think of the DNA testing as being just for ancestry and how the genetic links, and they’d be worried about things like that getting out. So, I think it’s making sure that they know that it’s kept confidential, it is only to do with medicines. It’s not going to cause something like that to get out.

Facilitator: [00:41:50] Pharmacist 9?

Pharmacist 9: [00:41:52] Uh, Pharmacist 3 touched on it there slightly. Um, I think patients’ understanding of the science is a huge hurdle. Um, you know, enzymes that help you process different medications and how that’s linked to your genes and how that links to what medicines are more suitable, is quite a scientifically complex thing to try and explain to people. Um, and then it’s kind of how the tests [00:42:22] would be done with the patients once they’ve kind of crossed that over. I suppose, um, whether that’s appropriate in the pharmacy or whether it’s postal or, you know, they do it themselves at home, or that kind of thing.

Facilitator: [00:42:37] Pharmacist 7 did you have question?

Pharmacist 7: [00:42:41] Um –

Facilitator: [00:42:42] A comment?

Pharmacist 7: [00:42:43] Yeah, just a comment. Um, I think maybe one of the concerns would be, um, just touching on what other people have said, is not damaging the relationship that we currently have with the GPs. Um, so just making sure that it’s just an understanding that we’re not trying to overtake their prescribing, we’re not trying to override what they’re saying, but it’s kind of like joint. So, that would have to be clearly established. Um, and I agree with like the dialogue as well. It’s explaining to patients. Um, I think the grey area, so mid-range, so where there’s either it’s [00:43:13] not that it doesn’t work completely well or that it’s, um, working fantastically, but that grey area, that mid-range. If things come back in that area, like what to do then. I think that’d be something to look at as well.

Facilitator: [00:43:26] Pharmacist 11? Uh, unmute yourself [Laughs].

Pharmacist 5: [00:43:34] OK. So, you mentioned limitations in the pharmacy, like practically, didn’t you? Because I was just thinking of two things, like space and time, which we are notoriously short of. Because some pharmacies, they struggle to carry out flu jabs because of not enough space. There’s hardly enough space to do an MUI. You’re sat not two metres away from somebody discussing their medicines, and they – if you brought out genome testing to them they’d probably think, “Why on earth have they said that? I’m in this really boxy room with them right now, and how are they going to carry this out?” It doesn’t, it doesn’t seem like it’s fit, [00:44:04] it doesn’t seem like it’s a provision that will be appropriate.

And the other one is time. Like, if you’re – if I was a patient going in for a pharmacogenomics testing, I’d want the person, the professional sat in front of me to be, um an expert who is going to go through it very methodically, very thoroughly and spend as much time with me as possible about this new, um, this new service, this new design of how we’re doing the medicines, and how we can adjust their medicine.

So, I, I mean, I’m thinking in my head it’s an, it’s an amazing idea [00:44:34] but then I’m thinking does everyone have 20 to 30 more, potentially more minutes to go through everything and make sure there’s not just communication but comprehension from the patient, that they understand what’s going on? Because otherwise, there is literally no point. I see what people mean, like is, is there any point? Some people want it, some people won’t understand.

Facilitator: [00:44:56] Thank you. Pharmacist 2? Unmute.

Pharmacist 2: [00:45:04] Yes, I totally, I totally agree with Pharmacist 11, the time is, is very important because that sort of service will require a lot of time and because to make them understand, uh, what, what the test is, even, even before starting the test. So, it can be quite challenging, especially if you’re running a very busy pharmacy. Yes, that would be a big challenge.

Facilitator: [00:45:29] Yeah, and I can see that, um, that time and space, um, uh, and maybe then some, some things within yourself about your own confidence and expertise to, to develop and offer such a service. Um, um, so what might we do to mitigate those? Um, because I completely understand that pharmacies are already busy and there’s, you know, strong, strong perception amongst patients that come into pharmacies that pharmacists are busy. So, what can we do? [00:45:59] Pharmacist 4?

Pharmacist 4: [00:46:01] Uh, we could, um – our job is to improve patient understanding, um, so we can use that to develop or improve patients’ health literacy. But also what we can do is maybe integrate this within, uh, within other services in the pharmacy such as NMS. So, while we’re doing the NMS, like Pharmacist 3 mentioned, if a medication’s not suitable, we could say, “OK, because you’ve tried these maybe let’s do this testing to see whether we can shed some more light on what’s more suitable for you.” So, yeah.

Pharmacist 5: [00:46:35] No, can I jump in? Like just because this service is so specific, I think tagging it onto the back of an NMS would be – I don’t know how – I, I mean, it, it’s good in theory, but I think it would have to be, “Oh, can you come back for this?” or, “Can I talk about this definitely with you?” because it’s so novel, it’s so – it’s quite intricate, and it’s personal. I feel like putting it with an NMS they wouldn’t take it as seriously as if you had it separately. So, I think it’d have to be to do with the staffing and (chuckles) [00:47:05] and trying to find people to cover while you’re sorting it out with, the managers who, um, maybe [unintelligible 00:47:11], but I think it –

Pharmacist 4: [00:47:14] I see where you’re coming from, but I think what we have (significant background noise) –

Facilitator: [00:47:19] So, carry on Pharmacist 4.

Pharmacist 4: [00:47:20] Yes. So, I, I see where Pharmacist 11 is coming from. There’s a lot of challenges within the pharmacy, and just to add another service on top of that is going to really press you in for time, but then again if you can delegate the tasks effectively, then the pharmacy [unintelligible 00:47:37] can have a technician, for example, that can take care of the dispensing and the checking and that not only frees up the time for the pharmacist to carry out these services, but I can also see that some pharmacies might be different and there’s challenges with regards [00:47:50] to that that needs to be addressed.

Facilitator: [00:47:54] OK. Uh, Pharmacist 9?

Pharmacist 9: [00:47:57] Um, we could ha- – rather than relying on the in-house pharmacist, um, they could get some kind of general knowledge on the topic and promote the service and there could be a video consultation with, uh, an expert to talk them through the results, um, who knows way more, and that means we get to do both. (Significant background noise) [Unintelligible [00:48:19] support the patient, and also have an expert to be able to share the information. I know it’s bringing in an extra [00:48:27] person, uh, but potentially, um, the in-house pharmacist could be involved in supporting that conversation with the patient as well, just, uh, kind of technology can aid that. And if there’s a person off site who has the time to devote to that person, because of the – if the service pays, enables that, then that’s potentially an, an option.

Facilitator: [00:48:53] Thank you. Um, so my last question is around, um, what would you need to enable a pharmacist led service like this to be offered? So, just thinking as broadly as you can, let’s imagine somebody said to you, “Right, we’re going to go for this. We, we’re going to try this as a, as an organisation,” what would you, you individually want or need to get stuck into it?

Pharmacist 1: [00:49:24] Good training I would say. We need to like be properly trained to carry out such a service because it’s going to be like – so, if you’re going to – if it’s going to cost X – um, if it’s going to be expensive I mean, then obviously, um, the patients expect a good service out of you as well. So, you should be like able to explain it to them in good language saying – telling them how – what, what you’re going to be doing and how it’s going to work for their benefit, [00:49:54] things like that. Yeah, feel confidence that you can provide that service and you know the patient will believe, believe in you and they would like be willing to take up the service.

Facilitator: [00:50:12] Thank you. And are there more – ? Is there anything else about training needs that you think ..? What, what sort of areas do you think you would need? I mean, we, we sprung on you that it’s pharmacogenomics, but, um, you know, is it something you, you’ve heard about? I know some of you have talked knowledgeably about it, but maybe some it’s a new idea. Um, what would it be that you’d be wanting to plug, plug this gap? Um, Pharmacist 2 you’ve got your hand up?

Pharmacist 2: [00:50:44] I think as well during, uh – uh, training is just at the beginning, of course, but as well to have like a sort of helpline that supports us during the service. So, any, any problem we may experience, we can still, uh, be supported by someone that, that knows because everything is new for us you know.

Facilitator: [00:51:07] Um, any other – ?

Pharmacist 3: [00:51:10] Also, a sort of cheat sheet as to what genes are more likely to affect – are more likely to be affected by medication, or rather which, which medications are more prone for certain genes so that we kno- – we can say, “OK.” For example, penicillin allergy. We’ve got to know which one, which genes are related to penicillin allergy by now. There’s a large number of people who say, “Oh, I’m allergic to penicillin,” [00:51:40] but they just have a rash. It’s not an actual full-blown anaphylaxis. It – you sort of say, “Yes, you, you may have an allergy, but you’ve got a mild allergy compared to this level of allergy.”

Facilitator: [00:51:59] Uh, Pharmacist 8 you’re on mute. Are you – ?

Pharmacist 8: [00:52:07] Oh, sorry, I was saying that – can you hear me?

Facilitator: [00:52:09] Yes, can now. Yes.

Pharmacist 8: [00:52:10] Uh. Most medications that are, you know, as I said, subject to genomic are long term things like alopurinol, warfarin, statins, clopidogrel, some antipsychotics. But acute medication subject to, say, the HLA-B gene, things like flucloxacillin, which are acute medications, I think you’d be, you’d be, um, between a rock and a hard place if you stopped a patient at, say, ten o’clock in the evening, you know, from taking their medications and I’m just going to make sure – I’m going to do a genomic test [00:52:40] and see if this is the – because presumably that’s where it may be of some benefit is in the few medications people are taking chronic medications are already – they know what their response is.

So, if you’re suggesting initiating medication, acute medication as Pharmacist 3 suggested, for maybe problems, side effects with – you’d need – you wouldn’t need backup, you’d need the ability to prescribe right then and there an alternative, ten o’clock in the evening with, with your child with [unintelligible 00:53:10] [00:53:10], you know penicillin. If you’re saying, “It’s not suitable for you child,” you need to say, “This is more suitable,” not “Can you go back to the out of hours.”

So, whatever it is you’re doing, if you’re going to stop or change medication based on whatever it is you’re doing, then you, you can’t just refer back. GPs don’t want – you know, all the primary care networks are saying, “We don’t want screening services and pharmacists that are driving traffic towards us. We want them to resolve it where they are,” which means you need to be an independent prescriber to say [00:53:40], you know – and have the GP let you decide based on what you’re going to do, you know, give them the right medication and for them not to be involved. So, autonomy would be the right, would be you know

Facilitator: [00:53:54] Um, and would, would you like some sort of clear pathway structure or guideline that helps to limit, you know, “This is where my involvement as a pharmacist begins and this is where my involvement ends?”

Pharmacist 5: [00:54:07] I was about to say that, because I was like, “It’s all well and good doing the background training and the theories and things like that, but we need the proper service training. So, clear aims, maybe an algorithm, actions, follow up, potential outcomes, the intended outcomes. We need to know what to do at every point in that consul-. I mean, we’re pharmacists , so we will know what to do, but sometimes like you need to know exactly which – like to pinpoint the algorithm where you are in it. So, if the patient gets this, you do this. If the patient gets that, [00:54:37] you do that. So, um, if they want any information from you they can always ask you, but really your job is quite simple at the end of the day. So, that’s what I was thinking anyway, do we need a structure?

Facilitator: [00:54:51] Um, and, um –

Female: [00:54:55] I think Pharmacist 10’s got her hand up Facilitator.

Facilitator: [00:54:57] Oh, sorry. Sorry, Pharmacist 10.

Pharmacist 10: [00:54:59] That’s all right. No problem. Um, for me, um, as I work in a busy pharmacy, um, it would be very important time, time spent for, um, personal training, um, to be able to deliver the service and also the precious time to spend with the patient, um, doing the service. Um, but also what I wanted to, um, to say, I’ve just read recently that new pharmacists are going to be trained as prescribers. [00:55:29] Um, so this is just maybe the beginning what we’re doing now, and because pharmacists will be prescriber in not as distant future, they can just use the pharmacogenomic, uh, testing to, um, to prescribe. I think that’s –

Facilitator: [00:55:48] It is indeed true, yes.

Pharmacist 10: [00:55:50] That’s, that’s it for me.

Facilitator: [00:55:54] That’s great. Thank you. Um, so my last question, it’s 9:01, I’ve got 60 seconds left. Um, if we were going to set this service up in a Day Lewis Pharmacy, what advice would you give us?

Pharmacist 3: [00:56:14] Start small.

Facilitator: [00:56:16] (Chuckles) Start small. Thank you. Pharmacist 9?

Pharmacist 5: [00:56:20] I would start with a few – oh, sorry (chuckles).

Pharmacist 9: [00:56:23] Um, start in Harrods where there’s some money to spend.

Facilitator: [00:56:25] (Laughs).

Pharmacist 1: [00:56:29] I think actually in Harrods they do have someone who’s doing it, or was going to start doing it.

Facilitator: [00:56:38] Uh, Pharmacist 11 you were going to say, sorry.

Pharmacist 5: [00:56:40] I was, I was going to say, uh, it shouldn’t be like a national roll out. They should select branches and then review them in maybe six months and see the progress, has it actually made a difference? Do pharmacists feel comfortable? Do patients actually benefit? Like, um, we’re talking about it now and it seems great, but then we need to have a review of the – maybe a like a pilot group and they should be selected based on the busyness of the store, um, just certain factors.

The space in the consultation room, the amount of people who go there for services anyway [00:57:10]. There’s certain things that, that it might do very well. The location, the proximity to the GP. So, I’m actually inside a GP surgery, which makes it very, very easy for me to walk over myself and talk to them. I’ve got a very good relationship with them, and that would put me in like great standing for this service.

But I know, um, in previous branches they’ve been on a high street in, um, really, really busy kind of retail environment, and it might not be, it might not be what they want genomic testing on the high street. They might not want that. They would probably seek the [00:57:40] one in the GP more than ones in other locations.

Pharmacist 3: [00:57:46] Also, figure out the minimum requirements. What is the minimum size of a consultation room that you’re going to need for this? What is the minimum amount of time that you’re expecting a pharmacist to spend with each patient? What is the minimum amount of time for the start, for the initial test, the minimum amount of time for the results of the test? So that we know what the bare minimum that we need to give is. So that we can see if we’ve got enough for the bare minimum.

Facilitator: [00:58:18] And the doing of the test, actually you would be collecting the sample, so that’s … you know, how do you feel about that?

Pharmacist 3: [00:58:32] During COVID I have done strep throat tests, I’ve done vaccinations. (Chuckles) I’m not too stressed on that front. Admittedly I PPE’d up to the max, but …

Facilitator: [00:58:49] So, any other comments? No, no. OK. Well, you’ve been great. You’ve been really, really good. You’ve had lots of different views, um, you’ve happily shared them, uh, amongst yourself. Um, really, really helpful. Um, this is the start of a process. This is just getting some, some groundings and there will be other things that will be happening, um, in, in the coming months. [00:59:19] But, um, this really does paint quite a nice picture of, of people’s understandings at this particular stage. So, thank you very much.

Thank you for your time, and I’m probably going to write to you a little bit later this week with some follow up. So, hopefully you won’t just ignore me when you see my name pop into your, your inboxes, but if you don’t have any other questions, thank you so much for your time [00:59:49] and Facilitator 2 I think we can stop recording now.
